# Supplementary material for: Visualization and design of the functional group distribution during statistical copolymerization
Source: Nat Commun. 2019 Aug 13;10:3641. doi: 10.1038/s41467-019-11368-6 (PMC6692376; doi:10.1038/s41467-019-11368-6)
Supplement: Supplementary file 1 — Supplementary Information [file 41467_2019_11368_MOESM1_ESM.pdf]

Supporting Information

Visualization and design of the functional group distribution during statistical copolymerization

Van Steenberge et al.

Supplementary Tables

Supplementary Table 1: Reactions and Arrhenius parameters for the cationic ring opening polymerization (CROP) of MeOx (*M_1*) and C2MestOx (*M_2*). In acetonitrile initiated by MeOTs (*I*) as well as the utilized rate coefficients at 140°C (reference temperature) and the corresponding monomer reactivity ratios *$r_{1/2}$*.

	Equation	*A* (L mol^{-1} s^{-1})	E_a (kJ mol^{-1})	*k* at 140°C (L mol^{-1} s^{-1})	Ref.
**Chain initiation**	$I + M_1 \xrightarrow{k_{i1}} P_{1,1} + X^-$	$A_{p11}/7.5$	75.4	1.94 10^{-2}	1
	$I + M_2 \xrightarrow{k_{i2}} P_{1,2} + X^-$	$A_{p22}/2$	63.0	8.28 10^{-2}	a
**Propagation**[b]	$P_{i,1} + M_1 \xrightarrow{k_{p11}} P_{i+1,1}$	5.00 10^{8}	75.4	1.45 10^{-1}	2
	$P_{i,2} + M_2 \xrightarrow{k_{p22}} P_{i+1,2}$	1.54 10^{7}	63.0	1.66 10^{-1}	3
	$P_{i,1} + M_2 \xrightarrow{k_{p12}} P_{i+1,2}$	A_{p11}/r_1	75.4	4.84 10^{-2}	a
	$P_{i,2} + M_1 \xrightarrow{k_{p21}} P_{i+1,1}$	A_{p22}/r_2	63.0	8.28 10^{-1}	a
**Chain transfer**	$P_{i,1} + M_1 \xrightarrow{k_{trM11}} D_{i,1} + P_{0,1}$	1.50 10^{7}	85.4	2.37 10^{-4}	1
	$P_{i,1} + M_2 \xrightarrow{k_{trM12}} D_{i,1} + P_{0,2}$	9.17 10^{6}	85.4	1.45 10^{-4}	a
	$P_{i,2} + M_1 \xrightarrow{k_{trM21}} D_{i,2} + P_{0,1}$	2.12 10^{6}	73.0	1.24 10^{-3}	a
	$P_{i,2} + M_2 \xrightarrow{k_{trM22}} D_{i,2} + P_{0,2}$	4.62 10^{5}	73.0	2.70 10^{-4}	a
**Macro propagation**	$P_{i,1} + D_{j,1} \xrightarrow{k_{pm11}} P_{i+j,1}$	$A_{p11}/100$	75.4	1.45 10^{-3}	1
	$P_{i,2} + D_{j,2} \xrightarrow{k_{pm22}} P_{i+j,2}$	$A_{p22}/100$	63.0	1.66 10^{-3}	c
	$P_{i,1} + D_{j,2} \xrightarrow{k_{pm12}} P_{i+j,2}$	$A_{p12}/100$	75.4	4.84 10^{-4}	c
	$P_{i,2} + D_{j,1} \xrightarrow{k_{pm21}} P_{i+j,1}$	$A_{p21}/100$	63.0	8.28 10^{-3}	c

[a]this work; [b]$i \geq 0$; [c]assumption as in ref 1.

Supplementary Table 2: Reactions and Arrhenius parameters for the cationic ring opening polymerization (CROP) of EtOx (M_1) and C3MestOx (M_2). In acetonitrile initiated by MeOTs (I) as well as the utilized rate coefficients at 140°C (reference temperature) and the corresponding monomer reactivity ratios $r_{1/2}$.

	Equation	A (L mol^{-1} s^{-1})	E_a (kJ mol^{-1})	k at 140°C (L mol^{-1} s^{-1})	Ref.
**Chain initiation**	$I + M_1 \xrightarrow{k_{i1}} P_{1,1} + X^-$	$A_{p11}/7.5$	73.4	1.38 10^{-2}	*a*
	$I + M_2 \xrightarrow{k_{i2}} P_{1,2} + X^-$	$A_{p22}/2$	71.7	8.20 10^{-2}	*a*
**Propagation**[b]	$P_{i,1} + M_1 \xrightarrow{k_{p11}} P_{i+1,1}$	1.99 10^{8}	73.4	1.04 10^{-1}	a
	$P_{i,2} + M_2 \xrightarrow{k_{p22}} P_{i+1,2}$	1.92 10^{8}	71.7	1.64 10^{-1}	3
	$P_{i,1} + M_2 \xrightarrow{k_{p12}} P_{i+1,2}$	A_{p11}/r_1	73.4	1.29 10^{-1}	*a*
	$P_{i,2} + M_1 \xrightarrow{k_{p21}} P_{i+1,1}$	A_{p22}/r_2	71.7	4.10 10^{-1}	*a*
**Chain transfer**	$P_{i,1} + M_1 \xrightarrow{k_{trM11}} D_{i,1} + P_{0,1}$	8.96 10^{6}	83.4	2.53 10^{-4}	*a*
	$P_{i,1} + M_2 \xrightarrow{k_{trM12}} D_{i,1} + P_{0,2}$	7.46 10^{6}	83.4	2.11 10^{-4}	*a*
	$P_{i,2} + M_1 \xrightarrow{k_{trM21}} D_{i,2} + P_{0,1}$	1.44 10^{7}	81.7	6.68 10^{-4}	*a*
	$P_{i,2} + M_2 \xrightarrow{k_{trM22}} D_{i,2} + P_{0,2}$	5.76 10^{6}	81.7	2.67 10^{-4}	*a*
**Macro propagation**	$P_{i,1} + D_{j,1} \xrightarrow{k_{pm11}} P_{i+j,1}$	$A_{p11}/100$	73.4	1.04 10^{-3}	*c*
	$P_{i,2} + D_{j,2} \xrightarrow{k_{pm22}} P_{i+j,2}$	$A_{p22}/100$	71.7	1.64 10^{-3}	*c*
	$P_{i,1} + D_{j,2} \xrightarrow{k_{pm12}} P_{i+j,2}$	$A_{p12}/100$	73.4	1.29 10^{-3}	*c*
	$P_{i,2} + D_{j,1} \xrightarrow{k_{pm21}} P_{i+j,1}$	$A_{p21}/100$	71.7	4.10 10^{-3}	*c*

[a]this work; [b]$i \geq 0$; [c]assumption as in ref 1.

Supplementary Table 3: Reactions and Arrhenius parameters for the cationic ring opening polymerization (CROP) of MeOx (*M_1*) and C3MestOx (*M_2*). In acetonitrile initiated by MeOTs (*I*) as well as the utilized rate coefficients at 140°C (reference temperature) and the corresponding monomer reactivity ratios $r_{1/2}$.

	Equation	A (L mol^{-1} s^{-1})	E_a (kJ mol^{-1})	k at 140°C (L mol^{-1} s^{-1})	Ref.
**Chain initiation**	$I + M_1 \xrightarrow{k_{i1}} P_{1,1} + X^-$	$A_{p11}/7.5$	75.4	1.94 10^{-2}	1
	$I + M_2 \xrightarrow{k_{i2}} P_{1,2} + X^-$	$A_{p22}/2$	71.7	8.20 10^{-2}	*a*
**Propagation**[b]	$P_{i,1} + M_1 \xrightarrow{k_{p11}} P_{i+1,1}$	5.00 10^{8}	75.4	1.45 10^{-1}	2
	$P_{i,2} + M_2 \xrightarrow{k_{p22}} P_{i+1,2}$	1.92 10^{8}	71.7	1.64 10^{-1}	3
	$P_{i,1} + M_2 \xrightarrow{k_{p12}} P_{i+1,2}$	A_{p11}/r_1	75.4	1.32 10^{-1}	*a*
	$P_{i,2} + M_1 \xrightarrow{k_{p21}} P_{i+1,1}$	A_{p22}/r_2	71.7	1.64	*a*
**Chain transfer**	$P_{i,1} + M_1 \xrightarrow{k_{trM11}} D_{i,1} + P_{0,1}$	1.50 10^{7}	85.4	2.37 10^{-4}	*1*
	$P_{i,1} + M_2 \xrightarrow{k_{trM12}} D_{i,1} + P_{0,2}$	4.45 10^{7}	85.4	6.46 10^{-4}	*a*
	$P_{i,2} + M_1 \xrightarrow{k_{trM21}} D_{i,2} + P_{0,1}$	8.64 10^{7}	81.7	4.01 10^{-3}	*a*
	$P_{i,2} + M_2 \xrightarrow{k_{trM22}} D_{i,2} + P_{0,2}$	5.76 10^{6}	81.7	2.67 10^{-4}	*a*
**Macro propagation**	$P_{i,1} + D_{j,1} \xrightarrow{k_{pm11}} P_{i+j,1}$	$A_{p11}/100$	75.4	1.45 10^{-3}	*c*
	$P_{i,2} + D_{j,2} \xrightarrow{k_{pm22}} P_{i+j,2}$	$A_{p22}/100$	71.7	1.64 10^{-3}	*c*
	$P_{i,1} + D_{j,2} \xrightarrow{k_{pm12}} P_{i+j,2}$	$A_{p12}/100$	75.4	1.32 10^{-3}	*c*
	$P_{i,2} + D_{j,1} \xrightarrow{k_{pm21}} P_{i+j,1}$	$A_{p21}/100$	71.7	1.64 10^{-2}	*c*

[a]this work; [b]$i \geq 0$; [c]assumption as in ref 1.

Supplementary Table 4: Reactions and Arrhenius parameters for the cationic ring opening polymerization (CROP) of EtOx (M_1) and C2MestOx (M_2). In acetonitrile initiated by MeOTs (I) as well as the utilized rate coefficients at 140°C (reference temperature) and the corresponding monomer reactivity ratios $r_{1/2}$.

	Equation	A (L mol^{-1} s^{-1})	E_a (kJ mol^{-1})	k at 140°C (L mol^{-1} s^{-1})	Ref.
**Chain initiation**	$I + M_1 \xrightarrow{k_{i1}} P_{1,1} + X^-$	$A_{p11}/7.5$	73.4	$1.38\ 10^{-2}$	*a*
	$I + M_2 \xrightarrow{k_{i2}} P_{1,2} + X^-$	$A_{p22}/2$	63.0	$8.28\ 10^{-2}$	*a*
**Propagation**[a]	$P_{i,1} + M_1 \xrightarrow{k_{p11}} P_{i+1,1}$	$1.99\ 10^{8}$	73.4	$1.04\ 10^{-1}$	a
	$P_{i,2} + M_2 \xrightarrow{k_{p22}} P_{i+1,2}$	$1.54\ 10^{7}$	63.0	$1.66\ 10^{-1}$	3
	$P_{i,1} + M_2 \xrightarrow{k_{p12}} P_{i+1,2}$	A_{p11}/r_1	73.4	$9.41\ 10^{-2}$	*a*
	$P_{i,2} + M_1 \xrightarrow{k_{p21}} P_{i+1,1}$	A_{p22}/r_2	63.0	$8.82\ 10^{-1}$	*a*
**Chain transfer**	$P_{i,1} + M_1 \xrightarrow{k_{trM11}} D_{i,1} + P_{0,1}$	$8.96\ 10^{6}$	83.4	$2.53\ 10^{-4}$	1
	$P_{i,1} + M_2 \xrightarrow{k_{trM12}} D_{i,1} + P_{0,2}$	$5.43\ 10^{6}$	83.4	$1.54\ 10^{-4}$	*a*
	$P_{i,2} + M_1 \xrightarrow{k_{trM21}} D_{i,2} + P_{0,1}$	$2.31\ 10^{6}$	73.0	$1.4\ 10^{-3}$	*a*
	$P_{i,2} + M_2 \xrightarrow{k_{trM22}} D_{i,2} + P_{0,2}$	$4.62\ 10^{5}$	73.0	$2.70\ 10^{-4}$	*a*
**Macro propagation**[e]	$P_{i,1} + D_{j,1} \xrightarrow{k_{pm11}} P_{i+j,1}$	$A_{p11}/100$	73.4	$1.04\ 10^{-3}$	*c*
	$P_{i,2} + D_{j,2} \xrightarrow{k_{pm22}} P_{i+j,2}$	$A_{p22}/100$	63.0	$1.66\ 10^{-3}$	*c*
	$P_{i,1} + D_{j,2} \xrightarrow{k_{pm12}} P_{i+j,2}$	$A_{p12}/100$	73.4	$9.41\ 10^{-4}$	*c*
	$P_{i,2} + D_{j,1} \xrightarrow{k_{pm21}} P_{i+j,1}$	$A_{p21}/100$	63.0	$8.82\ 10^{-3}$	*c*

[a]this work; [b]$i \geq 0$; [c]assumption as in ref 1.

Supplementary Table 5: Reactions and Arrhenius parameters for the cationic ring opening polymerization (CROP) of *n*PropOx (M_1) and C2MestOx (M_2). In acetonitrile initiated by MeOTs (I) as well as the utilized rate coefficients at 140°C (reference temperature) and the corresponding monomer reactivity ratios $r_{1/2}$.

	Equation	A (L mol^{-1} s^{-1})	E_a (kJ mol^{-1})	k at 140°C (L mol^{-1} s^{-1})	Ref.
**Chain initiation**	$I + M_1 \xrightarrow{k_{i1}} P_{1,1} + X^-$	$k_{p11,140°C}/7.5^c$	0^c	1.25 10^{-2}	*a*
	$I + M_2 \xrightarrow{k_{i2}} P_{1,2} + X^-$	$A_{p22}/2$	63.0	8.28 10^{-2}	*a*
**Propagation**[b]	$P_{i,1} + M_1 \xrightarrow{k_{p11}} P_{i+1,1}$	$k_{p11,140°C}{}^c$	0^c	9.35 10^{-2}	a
	$P_{i,2} + M_2 \xrightarrow{k_{p22}} P_{i+1,2}$	1.54 10^7	63.0	1.66 10^{-1}	3
	$P_{i,1} + M_2 \xrightarrow{k_{p12}} P_{i+1,2}$	$k_{p11,140°C}/r_1$	0^c	9.35 10^{-2}	*a*
	$P_{i,2} + M_1 \xrightarrow{k_{p21}} P_{i+1,1}$	k_{p22}/r_2	63.0	1.66	*a*
**Chain transfer**	$P_{i,1} + M_1 \xrightarrow{k_{trM11}} D_{i,1} + P_{0,1}$	$k_{trM11,140°C}{}^c$	0^c	9.35 10^{-5}	1
	$P_{i,1} + M_2 \xrightarrow{k_{trM12}} D_{i,1} + P_{0,2}$	$k_{trM12,140°C}{}^c$	0^c	4.21 10^{-4}	*a*
	$P_{i,2} + M_1 \xrightarrow{k_{trM21}} D_{i,2} + P_{0,1}$	6.93 10^5	73.0	4.05 10^{-4}	*a*
	$P_{i,2} + M_2 \xrightarrow{k_{trM22}} D_{i,2} + P_{0,2}$	4.62 10^5	73.0	2.70 10^{-4}	*a*
**Macro propagation**	$P_{i,1} + D_{j,1} \xrightarrow{k_{pm11}} P_{i+j,1}$	$A_{p11}/100$	0^c	9.35 10^{-4}	*d*
	$P_{i,2} + D_{j,2} \xrightarrow{k_{pm22}} P_{i+j,2}$	$A_{p22}/100$	63.0	1.66 10^{-3}	*d*
	$P_{i,1} + D_{j,2} \xrightarrow{k_{pm12}} P_{i+j,2}$	$A_{p12}/100$	0^c	9.35 10^{-4}	*d*
	$P_{i,2} + D_{j,1} \xrightarrow{k_{pm21}} P_{i+j,1}$	$A_{p21}/100$	63.0	1.66 10^{-2}	*d*

[a]this work; [b]$i \geq 0$; [c]formal description using E_a = 0 kJ mol^{-1} and $A = k$ due to only description of one isothermal experiment; [c]assumption as in ref 1.

Supplementary Table 6: Reactions and Arrhenius parameters for the cationic ring opening polymerization (CROP) of *n*PropOx (*M_1*) and C3MestOx (*M_2*). In acetonitrile initiated by MeOTs (*I*) as well as the utilized rate coefficients at 140°C (reference temperature) and the corresponding monomer reactivity ratios $r_{1/2}$.

	Equation	*A* (L mol^{-1} s^{-1})	E_a (kJ mol^{-1})	*k* at 140°C (L mol^{-1} s^{-1})	Ref.
**Chain initiation**	$I + M_1 \xrightarrow{k_{i1}} P_{1,1} + X^-$	$k_{p11,140°C}/7.5^c$	0^c	$1.25\ 10^{-2}$	a
	$I + M_2 \xrightarrow{k_{i2}} P_{1,2} + X^-$	$A_{p22}/2$	71.7	$8.20\ 10^{-2}$	a
**Propagation**^{b}	$P_{i,1} + M_1 \xrightarrow{k_{p11}} P_{i+1,1}$	$k_{p11,140°C}{}^c$	0^c	$9.35\ 10^{-2}$	a
	$P_{i,2} + M_2 \xrightarrow{k_{p22}} P_{i+1,2}$	$1.92\ 10^8$	71.7	$1.64\ 10^{-1}$	3
	$P_{i,1} + M_2 \xrightarrow{k_{p12}} P_{i+1,2}$	$k_{p11,140°C}/r_1$	0^c	$9.35\ 10^{-2}$	a
	$P_{i,2} + M_1 \xrightarrow{k_{p21}} P_{i+1,1}$	k_{p22}/r_2	71.7	$4.10\ 10^{-1}$	a
**Chain transfer**	$P_{i,1} + M_1 \xrightarrow{k_{trM11}} D_{i,1} + P_{0,1}$	$k_{trM11,140°C}{}^c$	0^c	$9.35\ 10^{-5}$	1
	$P_{i,1} + M_2 \xrightarrow{k_{trM12}} D_{i,1} + P_{0,2}$	$k_{trM12,140°C}{}^c$	0^c	$2.80\ 10^{-4}$	a
	$P_{i,2} + M_1 \xrightarrow{k_{trM21}} D_{i,2} + P_{0,1}$	$6.93\ 10^5$	81.7	$6.68\ 10^{-5}$	a
	$P_{i,2} + M_2 \xrightarrow{k_{trM22}} D_{i,2} + P_{0,2}$	$4.62\ 10^5$	81.7	$2.67\ 10^{-4}$	a
**Macro propagation**	$P_{i,1} + D_{j,1} \xrightarrow{k_{pm11}} P_{i+j,1}$	$A_{p11}/100$	0^c	$9.35\ 10^{-4}$	d
	$P_{i,2} + D_{j,2} \xrightarrow{k_{pm22}} P_{i+j,2}$	$A_{p22}/100$	71.7	$1.64\ 10^{-3}$	d
	$P_{i,1} + D_{j,2} \xrightarrow{k_{pm12}} P_{i+j,2}$	$A_{p12}/100$	0^c	$9.35\ 10^{-4}$	d
	$P_{i,2} + D_{j,1} \xrightarrow{k_{pm21}} P_{i+j,1}$	$A_{p21}/100$	71.7	$4.10\ 10^{-3}$	d

^{a}this work; $^b i \geq 0$; ^{c}formal description using E_a = 0 kJ mol^{-1} and $A = k$ due to only description of one isothermal experiment; ^{d}assumption as in ref 1.

Supplementary Table 7: Monomer reactivity ratios for propagation of 2-oxazolines at 140°C; *r* values; ratio of $k_{p,ii}$ to $k_{p,ij}$; $k_{p,ii/j}$: addition of cation with terminal monomer unit *i* to monomer *i/j*); *comonomer considered for FUNC-CLD calculation.

Cation/**crossM**[a]	**PhOx**	**C2MestOx***	**C3MestOx***	***i*PropOx**	**EtOx***	***n*PropOx**	***n*NonOx**	**MeOx***
PhOx	*1.0*	-	-	-	0.2[4,b(1)]	-	0.02[5]	0.03[1]
C2MestOx	-	*1.0*	-	-	0.2[3,c]	0.10 [c]	-	0.2[3,c]
C3MestOx	-	-	*1.0*	-	0.4[3,c]	0.40 [c]	-	0.1[3,c]
iPropOx	-	-	-	*1.0*	0.8[6,b(1)]	0.6[7,b(3)]	-	-
EtOx	7.9[4b]	1.1[3,c]	0.8[3,c]	1.8[6,b(2)]	*1.0*	1.0[8,7,b(3)]	1.1[9]	-
nPropOx	-	1.0[c]	1.0[c]	3.2[7,b(3)]	1.3[8,7,b(3)]	*1.0*	-	-
nNonOx	7.1[5]	-	-	-	1.1[9]	-	*1.0*	-
MeOx	10.0[10]	3.0[3,c]	1.1[3,c]	-	-	-	-	*1.0*

[a]monomer for cross-propagation, except at the diagonal; [b]not at 140 °C: (1)160 °C, (2) 42 °C, (3) 42 °C; [c]this work.

Supplementary Table 8: Chain transfer reactivity ratio trends for 2-oxazolines at 140°C; determined in the present work (r_{ct} values; ratio of $k_{trM,ii}$ to $k_{trM,ij}$; $k_{trM,ii/j}$: chain transfer involving cation with terminal monomer unit *i* and monomer *i/j*); *comonomer for FUNC-CLD calculation.

Cation/**Cross-M**[a]	**PhOx**	**C2MestOx***	**C3MestOx***	**EtOx***	***n*PropOx**	**MeOx***
PhOx	*1.0*	-	-	-	-	0
C2MestOx	-	*1.0*	-	0.2	0.67	0.22
C3MestOx	-	-	*1.0*	0.4	4.0	0.06
EtOx	-	1.65	1.2	*1.0*	-	-
*n*PropOx	-	0.22	0.3		*1.0*	-
MeOx	0[b]	1.6	0.37	-	-	*1.0*

[a] monomer for cross-chain transfer, except at the diagonal;[b] taken from ref. [1]

Supplementary Table 9: Scaling factor for X_n.

(co)monomer(s)	Scaling factor (-)
C2MestOx	0.80
C3MestOx	0.70
*n*PropOx	0.53
EtOx	0.50 or 1.05[a]
MeOx	0.47
MeOx-PhOx	0.55
MeOx-C2MestOx	0.65
MeOx-C3MestOx	0.60
EtOx-C2MestOx	0.67
EtOx-C3MestOx	0.75
*n*PropOx-C2MestOx	0.60
*n*PropOx-C3MestOx	0.85

[a] the eluent is chloroform and polystyrene standards were used for relative calculation of the molar mass and dispersity values, used in Supplementary Figure 5.

Supplementary Table 10 Effect of temperature and initial functional monomer amount on the fraction of non-functionalized chains ($f_{nonfunctionalized}$) and the coefficient of variation (C_v; Equation (1)). Gor CROP of MeOx and C2MestOx (total monomer concentration: 3 mol L^{-1}; solvent acetonitrile; target DP of 100; overall monomer conversion (X_m)=100%); color coding: Figure 7d in the main text (guide of the eye principle); related FUNC-CLDs Supplementary Figure 21.

Conditions	$C_{v,FUNCD}$ & NONFUNC (-)		
	140 °C	120 °C	100 °C
$f_{C2Mest0x,0}$=0.10	0.47 & 0.015	0.47 & 0.015	0.46 & 0.012
$f_{C2Mest0x,0}$=0.05	0.60 & 0.045	0.58 & 0.042	0.57 & 0.037

Supplementary Table 11. Overview of reactions and their rate coefficients at 110 °C, except termination, for modeling of atom transfer radical polymerization of styrene (M_1) with small amounts of N-propyl maleimide (M_2). 1-Bromoethyl benzene as ATRP initiator ($R_{0,1}X$), Cu(I) bromide/4,4'-dinonyl-2,2'-bipyridine as ATRP activator.

	Reaction step	k ((L mol^{-1})s^{-1})	Ref.
**Thermal initiation**			
Diels-Alder dimerization	$2M_1 \overset{k_d}{\rightarrow} D$	8.3×10^{-8}	21
Retro Diels-Alder dimerization	$D \overset{k_{dr}}{\longrightarrow} 2M_1$	9.5×10^{-5}	21
Molecule assisted hemolysis	$D + M_1 \overset{k_{thi}}{\longrightarrow} R_{0,2} + R_{0,3}$	4.1×10^{-8}	21
**Chain initiation**			
	$R_{0,y} + M_1 \overset{k_p^{0,1}}{\longrightarrow} R_1^1$	8.7×10^4	22
**Propagation**			
	$R_i^1 + M_1 \overset{k_p^{11}}{\longrightarrow} R_{i+1}^1$	8.7×10^3	21
	$R_i^1 + M_2 \overset{k_p^{12}}{\longrightarrow} R_{i+1}^2$	5.1×10^4	23
	$R_i^2 + M_1 \overset{k_p^{21}}{\longrightarrow} R_{i+1}^1$	5.8×10^4	23
	$R_i^2 + M_2 \overset{k_p^{22}}{\longrightarrow} R_{i+1}^2$	7.6×10^2	23
**Termination**[(d)]			
	$R_{0,y} + R_{0,z} \overset{k_{tc,app}^{0,y;0,z}}{\longrightarrow} R_0R_0$	composite	21,24
	$R_{0,y} + R_i^1 \overset{k_{tc,app}^{0,y;i}}{\longrightarrow} P_i$	k_t model	21,24
	$R_i^1 + R_j^1 \overset{k_{tc,app}^{i;j}}{\longrightarrow} P_{i+j}$		21,24
**Chain transfer**			
to monomer	$R_{0,y} + M_1 \overset{k_{trm}^{0,y}}{\longrightarrow} P_0 + R_{0,4}$	1.3×10^{-2}	21
	$R_i^1 + M_1 \overset{k_{trm}^{1}}{\longrightarrow} P_i + R_{0,4}$	1.3×10^{-2}	21
to dimer	$R_{0,y} + D \overset{k_{trd}^{0,y}}{\longrightarrow} P_0 + R_{0,3}$	1.2×10^2	21

table continued

	$R_i^1 + D \xrightarrow{k_{trd}^1} P_i + R_{0,3}$	1.2×10^2	21
**ATRP (de)activation**[(a)]			
Activation	$R_{0,1}X + M_y^nL_yX \xrightarrow{k_a^{0,1}} R_{0,1} + M_y^{n+1}L_yX_2$	2.1×10^{-2}	22
	$R_i^1X + M_y^nL_yX \xrightarrow{k_a^1} R_i^1 + +M_y^{n+1}L_yX_2$	2.1×10^{-2}	22
Deactivation	$R_{0,1} + M_y^{n+1}L_yX_2 \xrightarrow{k_{da}^{0,1}} R_{0,1}X + M_y^nL_yX$	2.1×10^{8}	(b)
	$R_i^1 + M_y^{n+1}L_yX_2 \xrightarrow{k_{da}^1} R_i^1X + M_y^nL_yX$	2.1×10^{8}	(b)

[(a)]Activation/deactivation of R_i^2X/R_i^2 can be neglected on related work on nitroxide mediated polymerization;[23] (b) this work

Supplementary Table 12. Model validation for the first ATRP case. Experimental data from Pfeifer and Lutz;[20] 1 eq. of maleimide monomer (with respect to the initial styrene amount) is added at a monomer conversion of 35%; target DP of 100; [styrene]$_0$:[R$_0$X]$_0$:[Activator]$_0$: 100:1:1; 110°C; values in brackets are the simulated values.

Time (min)	Conv M_1 (-)	Conv M_2 (-)
108	0.39 (0.38)	0.75 (0.80)
138	0.42 (0.44)	>0.99 (>0.99)
1380	0.80 (0.90)	>0.99 (>0.99)

Supplementary Table 13. Overview of reactions and their rate coefficients at 70 °C, except termination, for modeling of bulk atom transfer radical polymerization of 2-ethylhexyl acrylate (M_1) and glycidyl methacrylate (M_2); methyl 2-bromo propionate as ATRP initiator ($R_{0,1}X$), Cu(I) chloride/2,2'-bipyridine as ATRP activator.

	Reaction step	k ((L mol^{-1})s^{-1})	Ref.
**Chain initiation**			
	$R_0 + M_1 \xrightarrow{k_p^{0,1}} R_1^1$	3.6×10^4	(a)
	$R_0 + M_2 \xrightarrow{k_p^{0,2}} R_1^2$	1.0×10^5	(a)
**Propagation**			
	$R_i^1 + M_1 \xrightarrow{k_p^{11}} R_{i+1}^1$	3.6×10^4	27
	$R_i^1 + M_2 \xrightarrow{k_p^{12}} R_{i+1}^2$	1.0×10^5	28
	$R_i^2 + M_1 \xrightarrow{k_p^{21}} R_{i+1}^1$	4.0×10^2	28
	$R_i^2 + M_2 \xrightarrow{k_p^{22}} R_{i+1}^2$	1.1×10^3	29
**Termination[(d)]**			
	$R_{0,} + R_0 \xrightarrow{k_{tc,app}^{0,y;0,z}} R_0R_0$	composite	24
	$R_0 + R_i^k \xrightarrow{k_{tc,app}^{0,y;i}} P_i$	k_t model	24
	$R_i^k + R_j^l \xrightarrow{k_{tc,app}^{i;j}} P_{i+j}$		24
**ATRP (de)activation**			
Activation	$R_{0,1}X + M_y^nL_yX \xrightarrow{k_a^{0,1}} R_{0,1} + M_y^{n+1}L_yX_2$	1.5×10^{-2}	(a)
	$R_i^1X + M_y^nL_yX \xrightarrow{k_a^1} R_i^1 + M_y^{n+1}L_yX_2$	1.5×10^{-2}	(b)
	$R_i^2X + M_y^nL_yX \xrightarrow{k_a^2} R_i^2 + M_y^{n+1}L_yX_2$	$10 \times k_a^1$	(a)
Deactivation	$R_{0,1} + M_y^{n+1}L_yX_2 \xrightarrow{k_{da}^{0,1}} R_{0,1}X + M_y^nL_yX$	3.0×10^7	(a)
	$R_i^1 + M_y^{n+1}L_yX_2 \xrightarrow{k_{da}^1} R_i^1X + M_y^nL_yX$	3.0×10^7	(b)
	$R_i^2 + M_y^{n+1}L_yX_2 \xrightarrow{k_{da}^1} R_i^2X + M_y^nL_yX$	$0.1 \times k_{da}^1$	(a)

(a) For simplicity taken equal to value rate coefficient macrospecies; (b) this work

Supplementary Table 14. Model validation for the second ATRP case. Experimental data from ref 28; target DP of 100; $[M]_0$:$[R_0X]_0$:$[Activator]_0$: 100:1:1; 70°C; value in brackets are the simulated values; first column: initial feed composition.

M_1 vs. M_2	Time (min)	Conv. (-)	Dispersity	M_1 conv. (-)	M_2 conv. (-)
80:20	180	0.71 (0.75)	1.45 (1.53)	0.78 (0.81)	0.22 (0.19)
60:40	150	0.43 (0.50)	1.45 (1.49)	0.84 (0.75)	0.16 (0.19)
40:60	300	0.46 (0.60)	1.38 (1.29)	0.57 (0.65)	0.43 (0.56)
30:70	140	0.57 (0.52)	1.47 (1.41)	0.43 (0.51)	0.58 (0.52)
20:80	300	0.61 (0.64)	1.54 (1.32)	0.26 (0.24)	0.74 (0.70)

Supplementary Table 15. Effect of target DP on NON-FUNC value for "perfect" (so theoretical) functionalization chemistry; 100% conversion; variation of initial amount of functional comonomer; generated based on Supplementary Table 1 but without side reactions.

Target DP	50 mol%	10 mol%	5 mol%
25	0.00076	0.099	0.30
50	0.00041	0.015	0.098
100	0.00027	0.0025	0.014
200	0.00024	0.0018	0.0032
400	0.00040	0.0017	0.0025

Supplementary Table 16: Summary of effect of reaction parameters on the fraction of non-functionalized chains ($f_{\text{nonfunctionalized}}$) and the coefficient of variation ($C_{v,FUNCD}$; Equation (2)). For CROP of a functional and a non-functional monomer (total monomer concentration: 3 mol L^{-1}; solvent acetonitrile; overall monomer conversion (X_m)=100%); color coding: Figure 7d in the main text ("bad" to "excellent" functionalization: dark red to dark green, with intermediate colors orange, yellow and light green; FUNCDs constructed out of FUNC-CLDs from this section; model-based design allows to determine optimal conditions for a given target DP: highlighted in bold for this range of parameters.

Reaction conditions		$C_{v,FUNCD}$ & $f_{\text{nonfunctionalized}}$ (-)	
		**MeOx/C2MestOx**	EtOx/C2MestOx
1	$f_{C2Mest0x,0}$=0.02; target DP=100; T=100°C	0.82 & 0.20	0.82 & 0.21
2	$f_{C2Mest0x,0}$=0.02; target DP=100; T=140°C	0.82 & 0.20	0.85 & 0.24
3	$f_{C2Mest0x,0}$=0.02; target DP=400; T=100°C	0.55 & 0.072	0.75 & 0.11
4	$f_{C2Mest0x,0}$=0.02; target DP=400; T=140°C	0.60 & 0.11	0.82 & 0.14
5	$f_{C2Mest0x,0}$=0.10; target DP=100; T=100°C	0.46 & 0.015	0.46 & 0.018
6	$f_{C2Mest0x,0}$=0.10; target DP=100; T=140°C	0.47 & 0.02	0.49 & 0.026
7	$f_{C2Mest0x,0}$=0.10; target DP=400; T=100°C	0.51 & 0.012	0.62 & 0.015
8	$f_{C2Mest0x,0}$=0.10; target DP=400; T=140°C	0.59 & 0.017	0.70 & 0.020
9	**$f_{C2Mest0x,0}$=0.13; *target DP=100*; T=100°C**	**0.39 & 0.008**	0.43 & 0.011
10	$f_{C2Mest0x,0}$=0.13; target DP=100; T=140°C	0.45 & 0.012	0.47 & 0.016
11	**$f_{C2Mest0x,0}$=0.13; *target DP=400*; T=100°C**	**0.49 & 0.008**	0.61 & 0.008
12	$f_{C2Mest0x,0}$=0.13; target DP=400; T=140°C	0.58 & 0.013	0.69 & 0.013

Supplementary Figures

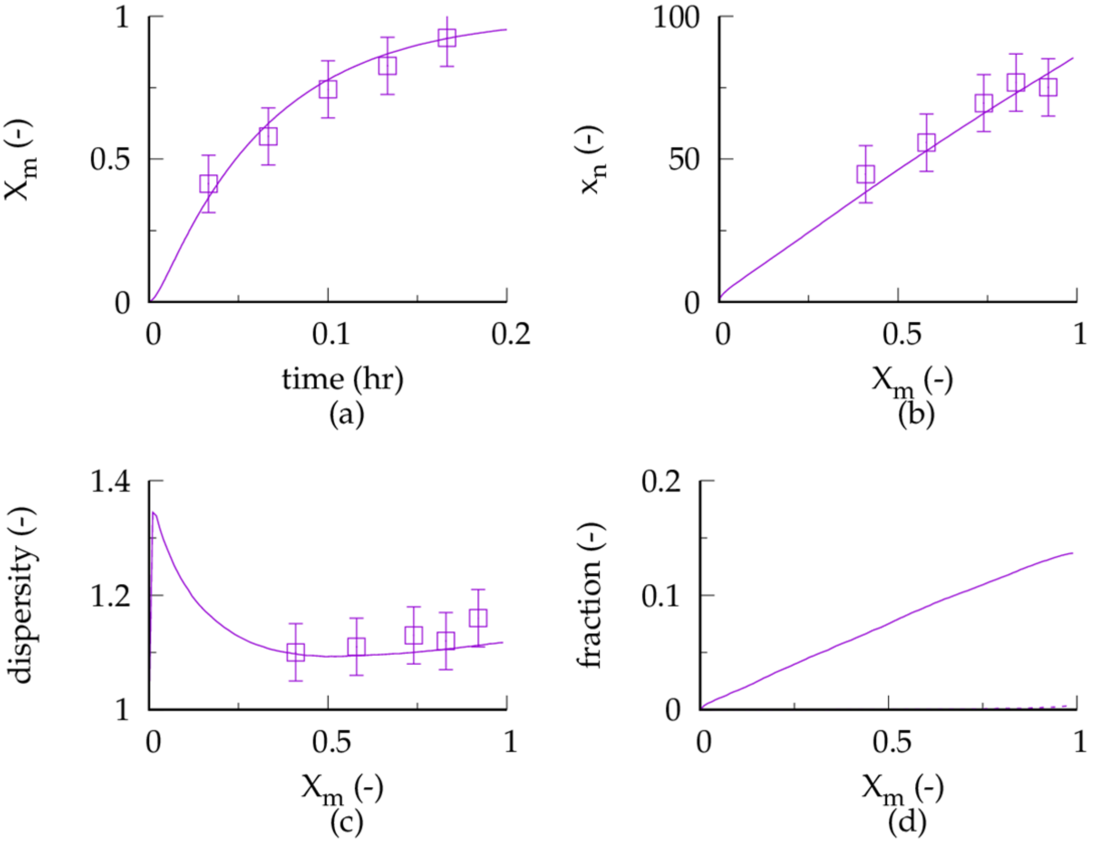

Supplementary Figure 1: Model validation homopolymerization 1. (a) Monomer conversion profile; (b) number average chain length (x_n); (c) dispersity; (d) macromonomer fraction (bottom, dashed) and fraction of branched chains (top, dashed) as a function of overall monomer conversion (X_m) for the CROP of MeOx. Reaction conditions: $[M[_0 = 3$ mol L^{-1} (solvent acetonitrile); target DP = 100, T = 140°C. Experimental data from Bouten *et al.*[3]; the reported error bars relate to the standard deviations following from repeat experiments.

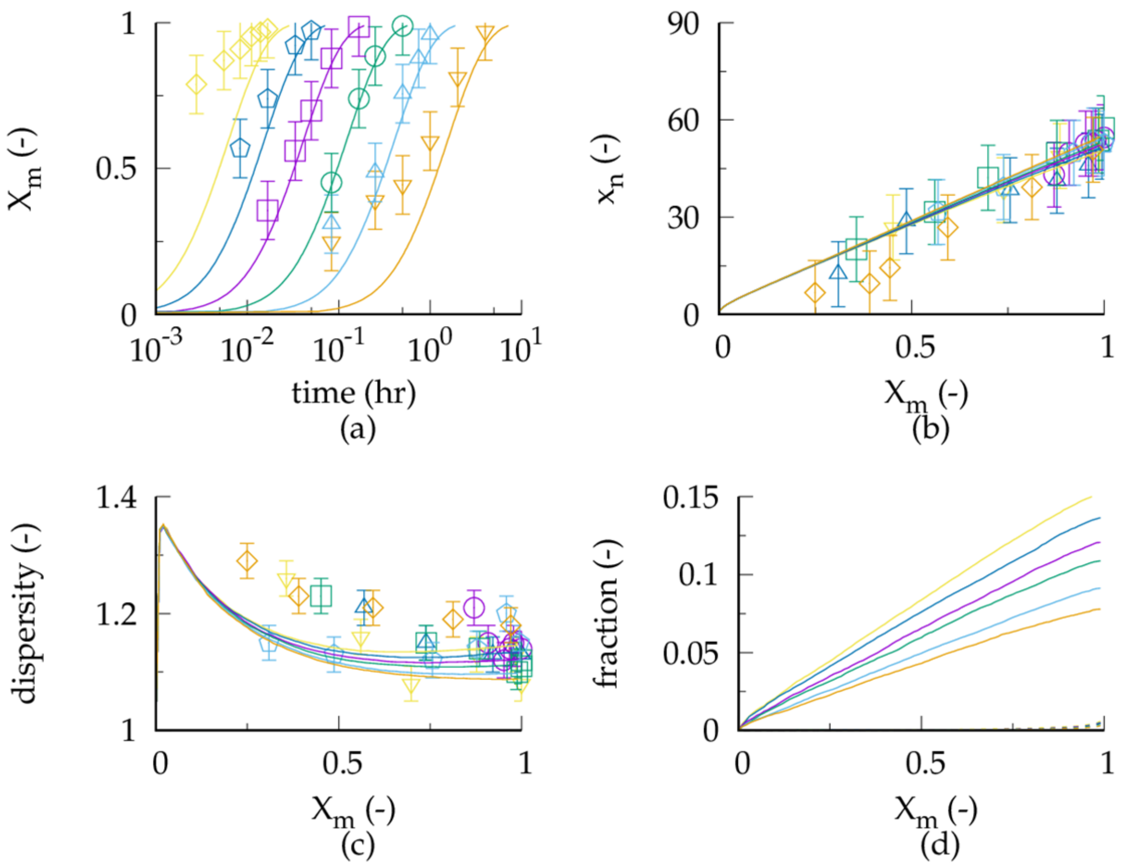

Supplementary Figure 2: Model validation homopolymerization 2. (a) Monomer conversion profile; (b) number average chain length (x_n); (c) dispersity; (d) macromonomer fraction (bottom, dashed) and fraction of branched chains (top, dashed) as a function of overall monomer conversion (X_m) for the CROP of EtOx; $[M[_0$ = 4 mol L^{-1} (solvent acetonitrile); target DP = 60, Temperature = 80 (orange), 100 (light blue), 120 (green), 140 (purple), 160 (blue), 180 °C (yellow), experimental data from Wiesbrock et al.[11]

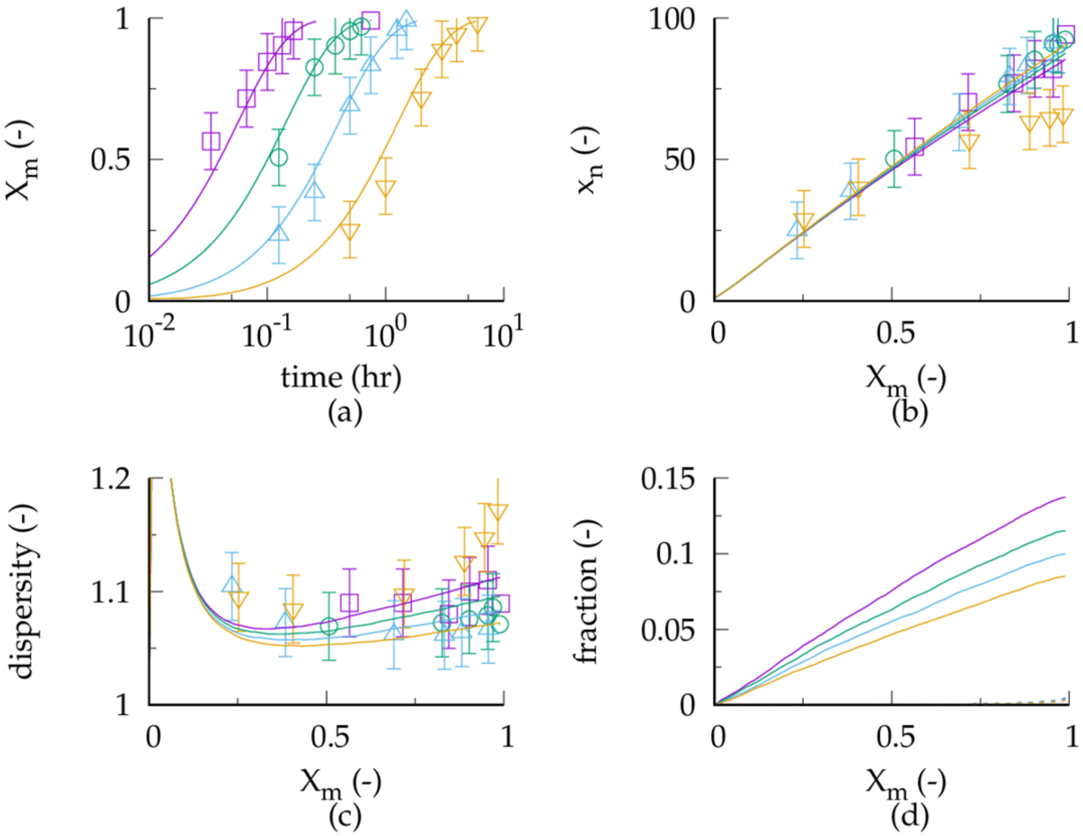

Supplementary Figure 3: Model validation homopolymerization 3. (a) Monomer conversion profile; (b) number average chain length (x_n); (c) dispersity; (d) macromonomer fraction (bottom, dashed) and fraction of branched chains (top, dashed) as a function of overall monomer conversion (X_m) for the CROP of C2MestOx. $[M[_0 = 3$ mol L^{-1} (solvent acetonitrile); target DP = 100, Temperature = 80 (orange), 100 (blue), 120 (green), 140°C (purple), experimental data from Bouten *et al.* [3]; the reported error bars relate to the standard deviations following from repeat experiments.

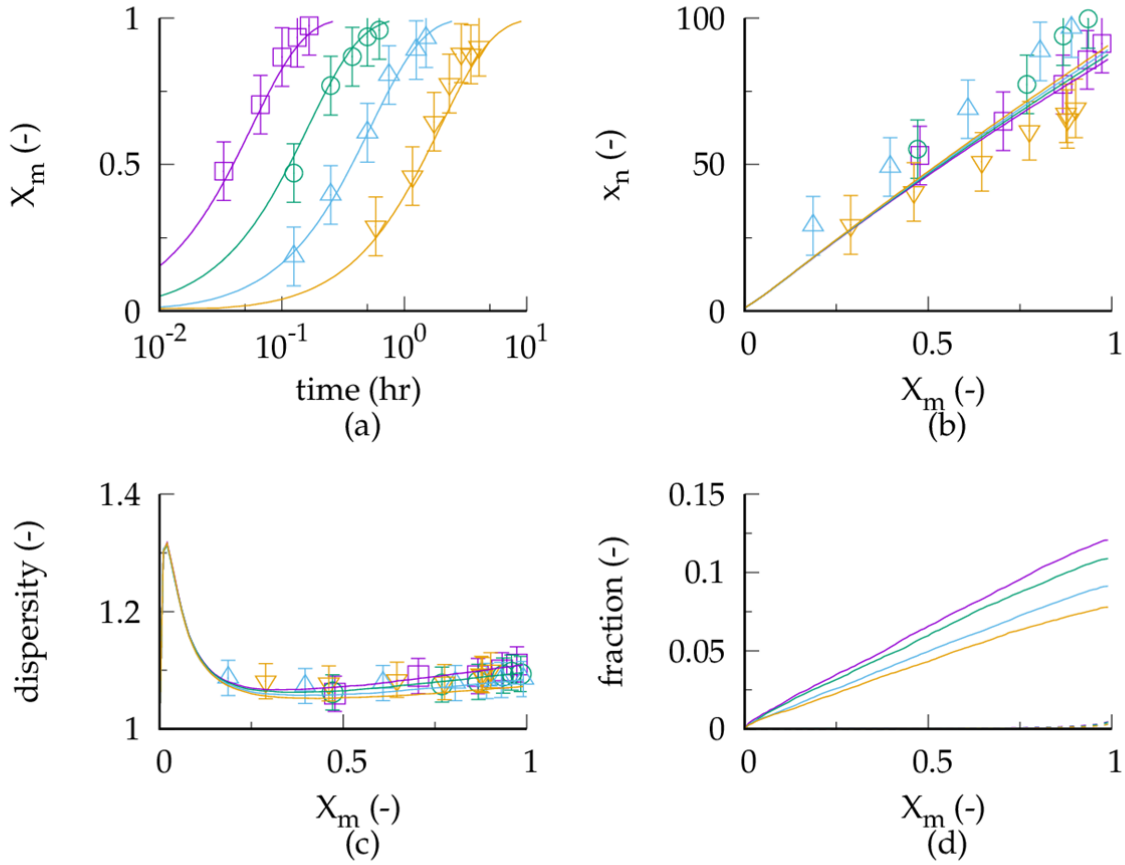

Supplementary Figure 4: Model validation homopolymerization 4. (a) Monomer conversion profile; (b) number average chain length (x_n); (c) dispersity; (d) macromonomer fraction (bottom, dashed) and fraction of branched chains (top, dashed) as a function of overall monomer conversion (X_m) for the CROP of C3MestOx. $[M[_0 = 3$ mol L^{-1} (solvent acetonitrile); target DP = 100, T = 80 (orange), 100 (blue), 120 (green), 140°C (purple), experimental data from Bouten et al.[3] ; the reported error bars relate to the standard deviations following from repeat experiments.

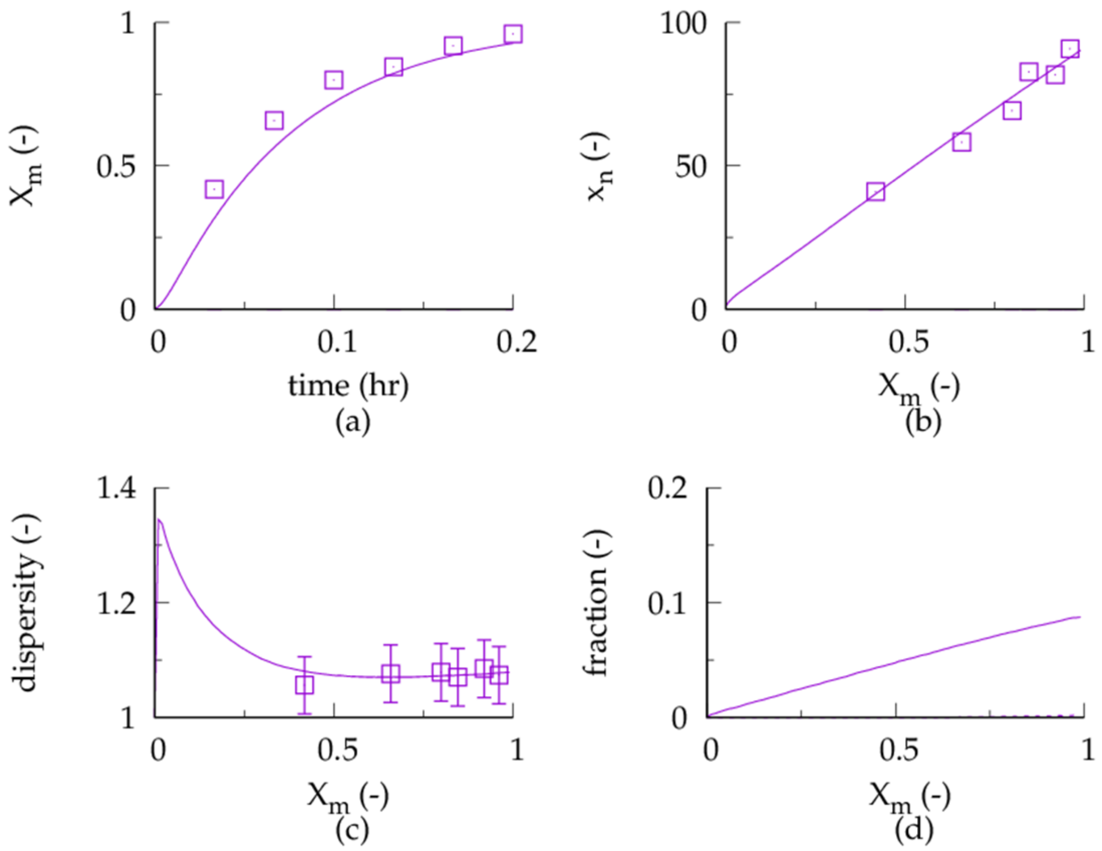

Supplementary Figure 5: Model validation homopolymerization 5. (a) Monomer conversion profile; (b) number average chain length (x_n); (c) dispersity; (d) macromonomer fraction (bottom, dashed) and fraction of branched chains (top, dashed) as a function of overall monomer conversion (X_m) for the CROP of nPropOx. $[M[_0 = 4$ mol L^{-1} (solvent acetonitrile);, target DP = 100, Temperature = 140°C, experimental data from Bouten *et al.*[3]; the reported error bars relate to the standard deviations following from repeat experiments.

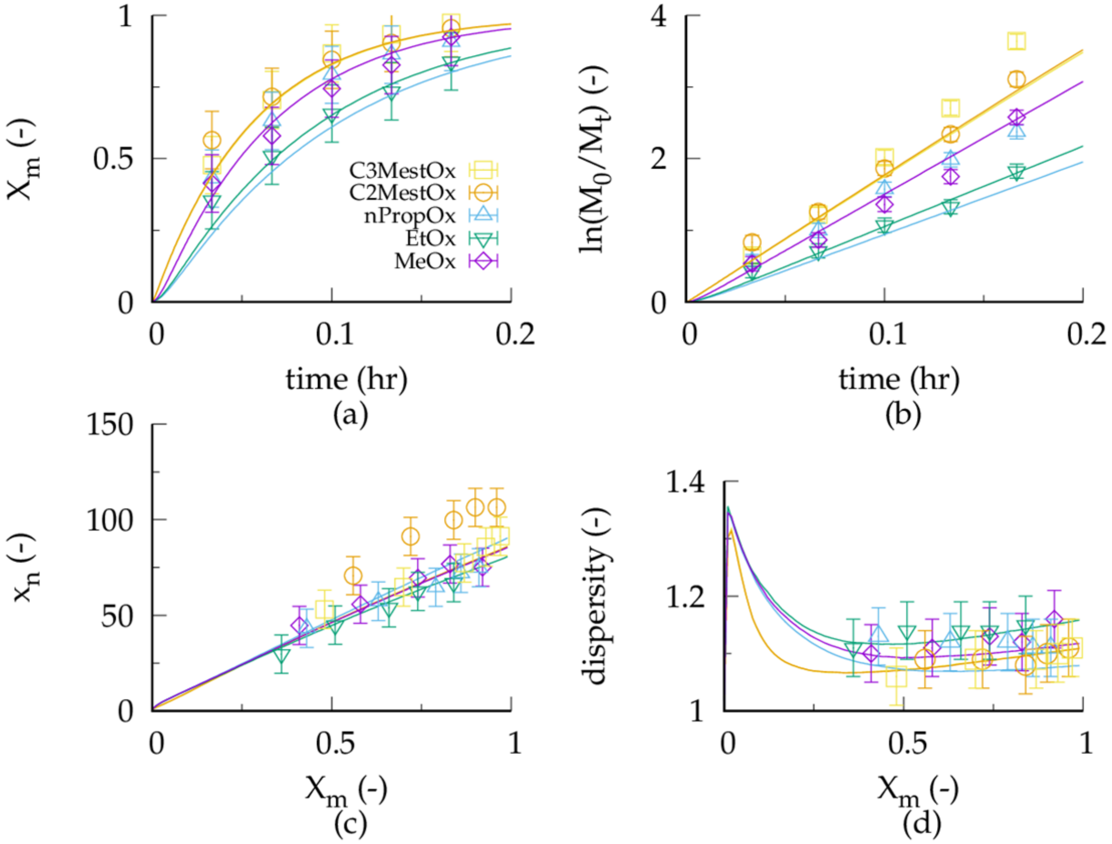

Supplementary Figure 6: Model validation homopolymerization 6. (a) Monomer conversion profile; (b) first-order kinetic plot (x_n); (c) number average chain length; (d) dispersity; for the CROP of different 2-oxazoline monomers. [M[$_0$ = 3 mol L^{-1} (solvent acetonitrile);, target DP = 100, Temperature = 140°C, experimental data from Bouten *et al.*[3]; the reported error bars relate to the standard deviations following from repeat experiments.

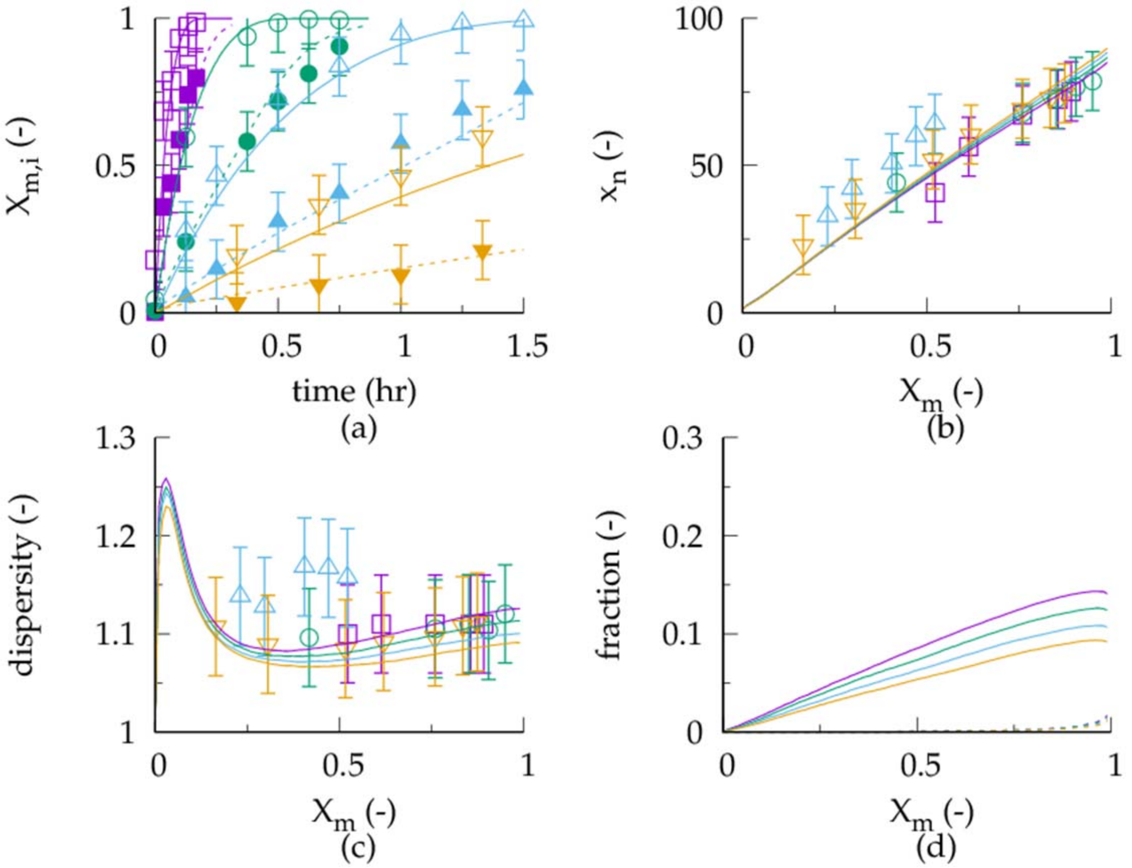

Supplementary Figure 7 (also Figure 3 in the main text): Model validation copolymerization 1. (a) Comonomer conversion profiles with C2MestOx (closed symbols) slower consumption than MeOx (open symbols).; (b) number average chain length (x_n); (c) dispersity; (d) macromonomer fraction (bottom; dashed) and fraction of branched chains (top; dashed) as a function of overall monomer conversion (X_m) for CROP of MeOx and C2MestOx under equimolar conditions (total monomer concentration: 3 mol L^{-1}; solvent acetonitrile; target DP = 100; 80 (orange), 100 (blue), 120 (green), and 140 °C (purple)); lines: simulations; symbols: experimental data.[3]; the reported error bars relate to the standard deviations following from repeat experiments.

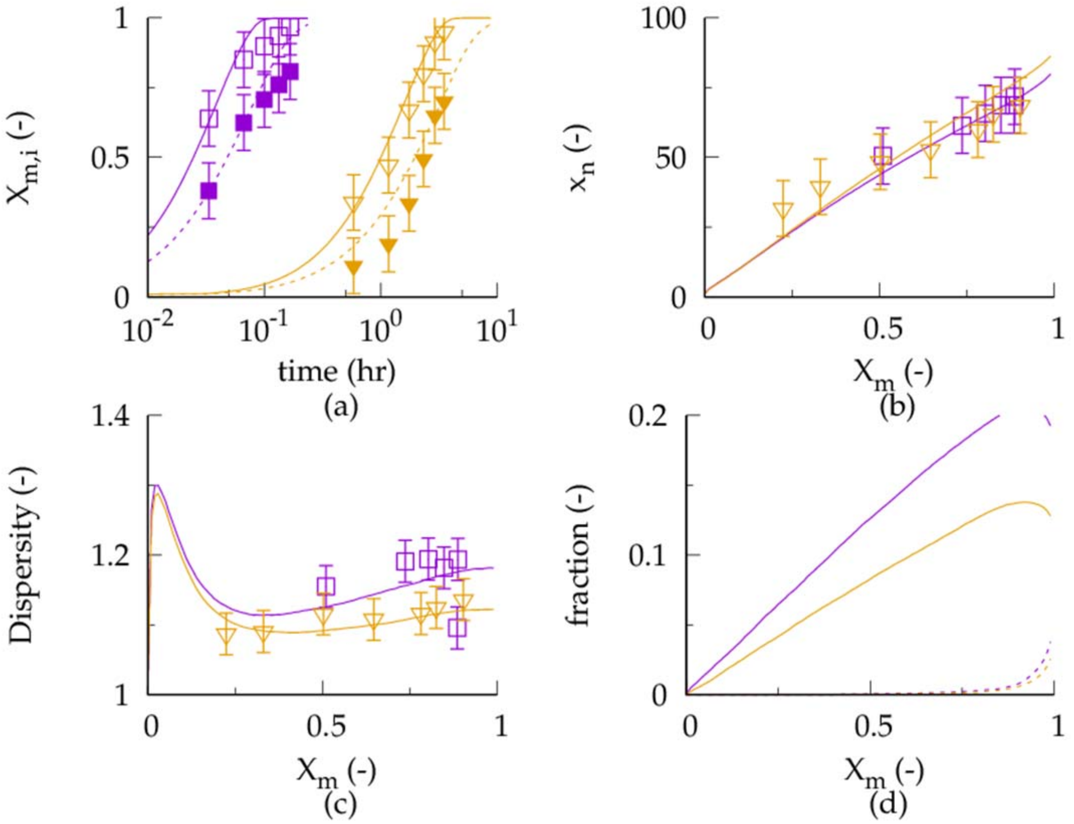

Supplementary Figure 8: Model validation copolymerization 2. (a) Comonomer conversion profiles; (b) number average chain length (x_n); (c) dispersity; (d) macromonomer fraction (bottom; dashed) and fraction of branched chains (top; dashed) as a function of overall monomer conversion (X_m) for CROP of MeOx and C3MestOx under equimolar conditions (total monomer concentration M_0 = 3 mol L^{-1}, target DP = 100, Temperature = 140 (purple) and 80 °C (orange). Experimental data from Bouten *et al.*[3]; the reported error bars relate to the standard deviations following from repeat experiments.

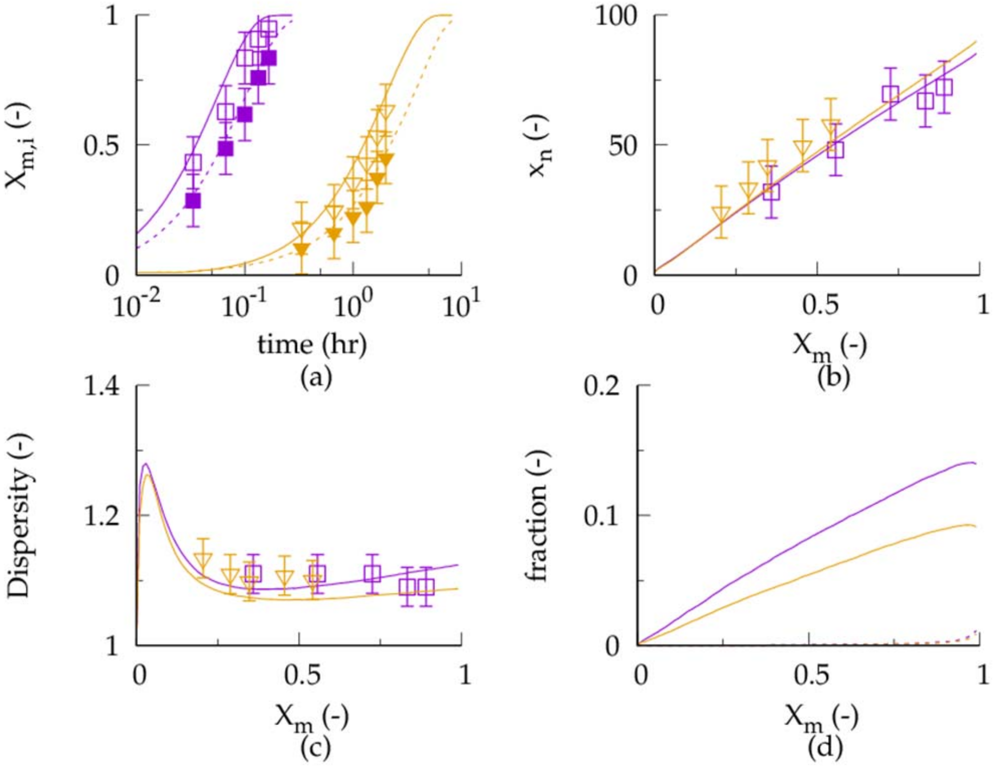

Supplementary Figure 9: Model validation copolymerization 3. (a) Comonomer conversion profiles; (b) number average chain length (x_n); (c) dispersity; (d) macromonomer fraction (bottom; dashed) and fraction of branched chains (top; dashed) as a function of overall monomer conversion (X_m) for CROP of EtOx and C2MestOx under equimolar conditions (total monomer concentration $[M]_0$ = 3 M, target DP = 100, solvent: acetonitrile; Temperature = 140 (purple) and 80 °C (orange). Experimental data from Bouten *et al.*[3]; the reported error bars relate to the standard deviations following from repeat experiments.

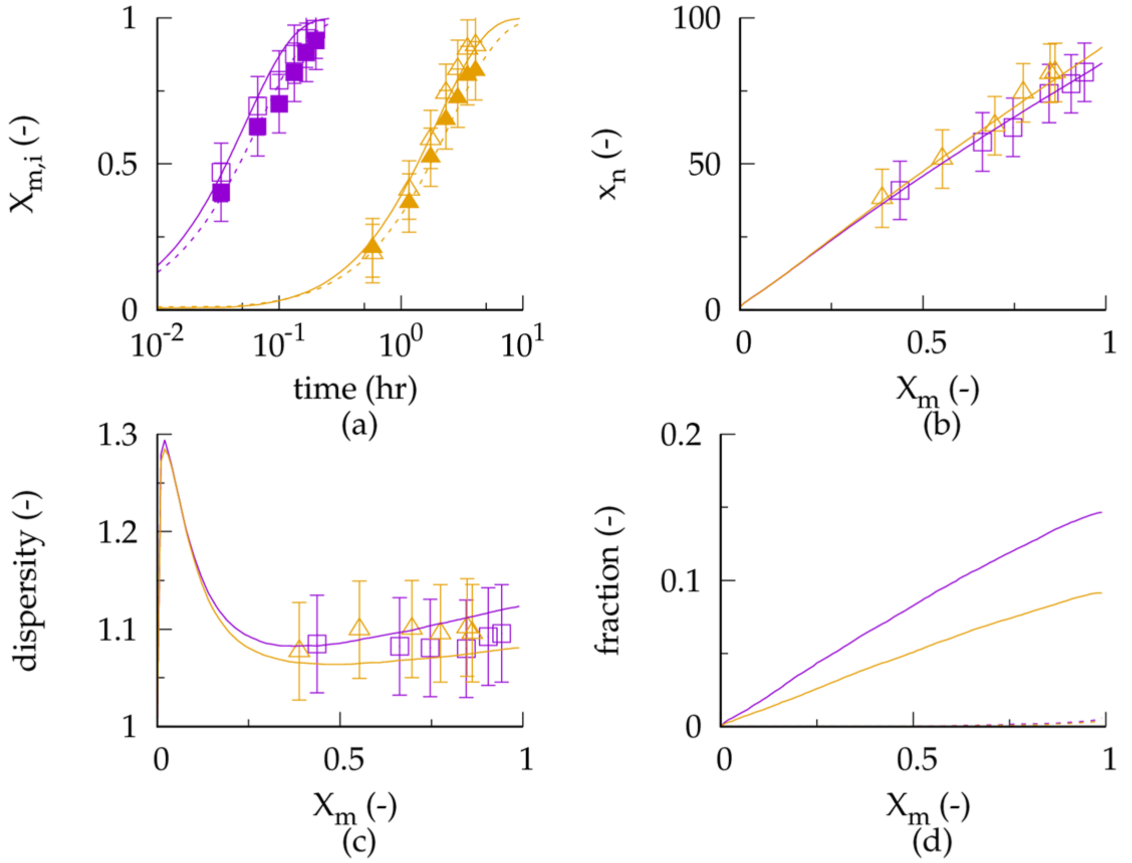

Supplementary Figure 10: Model validation copolymerization 4. (a) Comonomer conversion profiles; (b) number average chain length (x_n); (c) dispersity; (d) macromonomer fraction (bottom; dashed) and fraction of branched chains (top; dashed) as a function of overall monomer conversion (X_m) for CROP of EtOx and C3MestOx under equimolar conditions (total monomer concentration $[M]_0$ = 3 mol L^{-1}, target DP = 100, Temperature = 140 (purple) and 80 °C (orange). Experimental data from Bouten *et al.*[3]; solvent: acetonitrile; ; the reported error bars relate to the standard deviations following from repeat experiments.

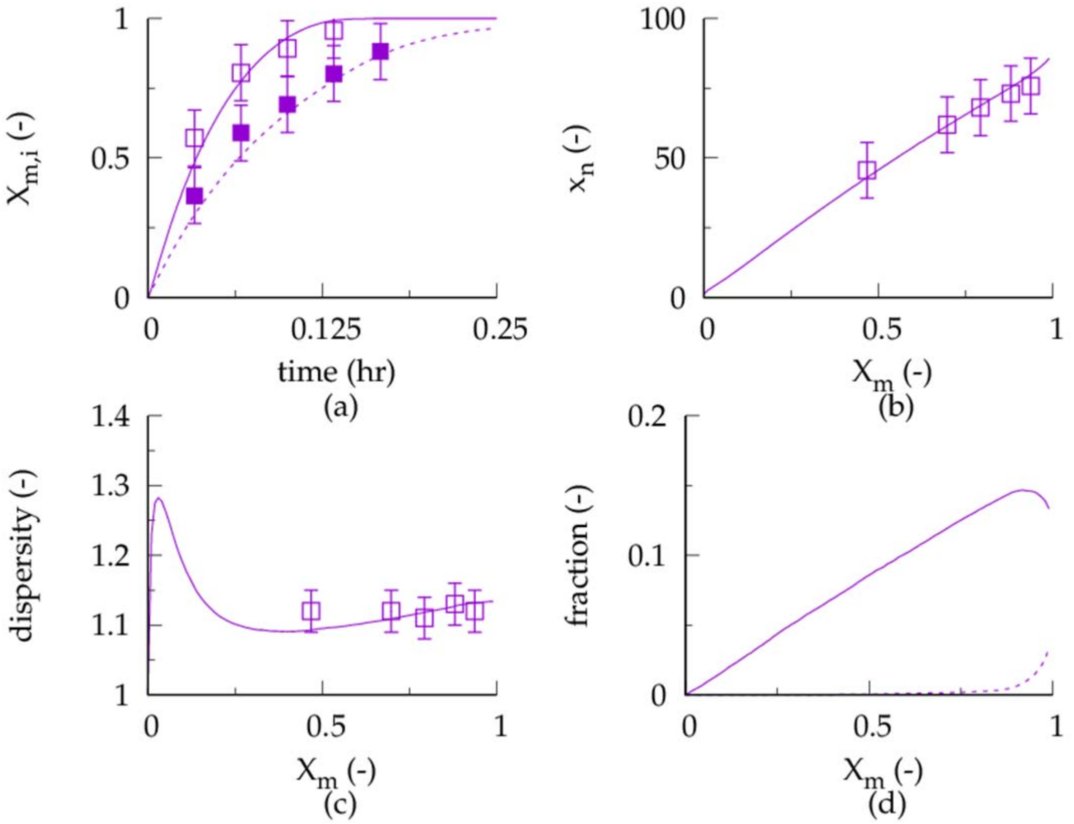

Supplementary Figure 11: Model validation copolymerization 5. (a) Comonomer conversion profiles; (b) number average chain length (x_n); (c) dispersity; (d) macromonomer fraction (bottom; dashed) and fraction of branched chains (top; dashed) as a function of overall monomer conversion (X_m) for CROP of nPropOx and C2MestOx under equimolar conditions (total monomer concentration $[M]_0$ = 3 mol L^{-1}, target DP = 100, Temperature = 140°C; experimental data from Bouten *et al.*[3]**; solvent: acetonitrile; ; the reported error bars relate to the standard deviations following from repeat experiments.**

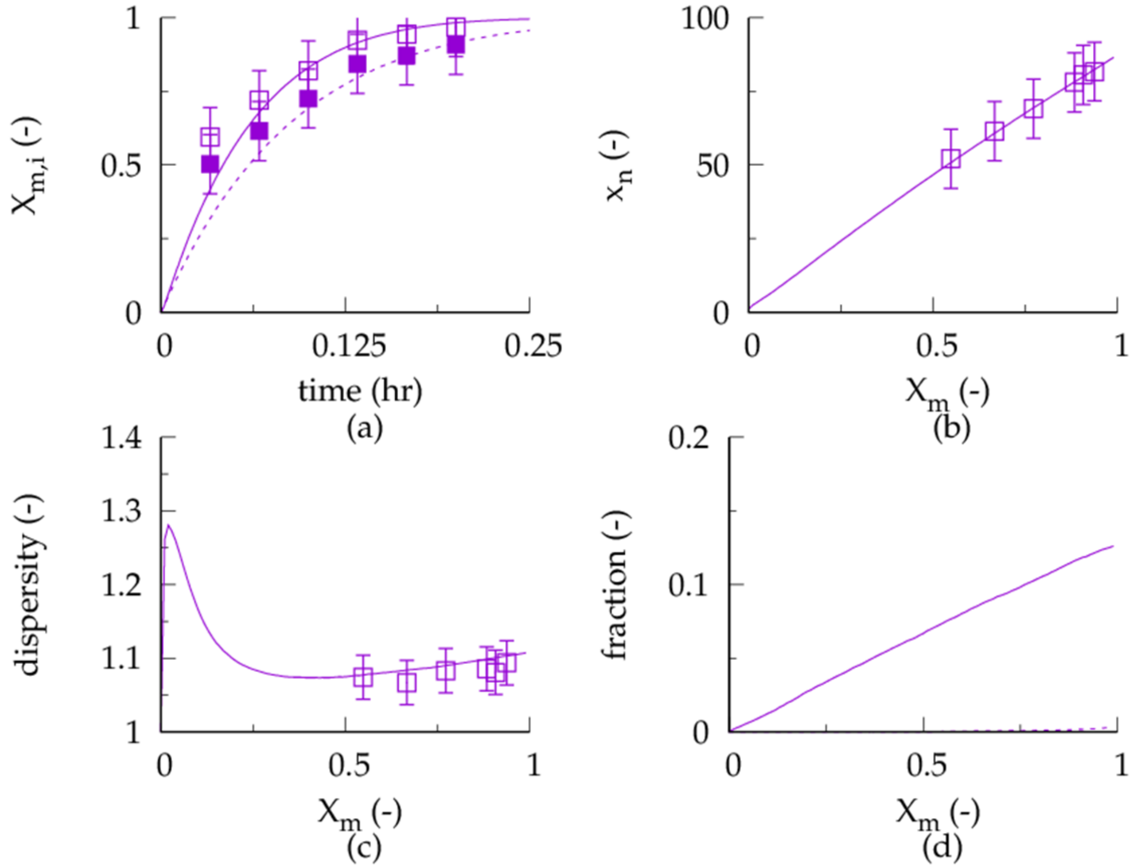

Supplementary Figure 12: Model validation copolymerization 6. (a) Comonomer conversion profiles; (b) number average chain length (x_n); (c) dispersity; (d) macromonomer fraction (bottom; dashed) and fraction of branched chains (top; dashed) as a function of overall monomer conversion (X_m) for CROP of nPropOx and C3MestOx under equimolar conditions (total monomer concentration $[M]_0 = 3$ mol L^{-1}, target DP = 100, Temperature = 140°C; experimental data from Bouten *et al.*[3]; the reported error bars relate to the standard deviations following from repeat experiments.

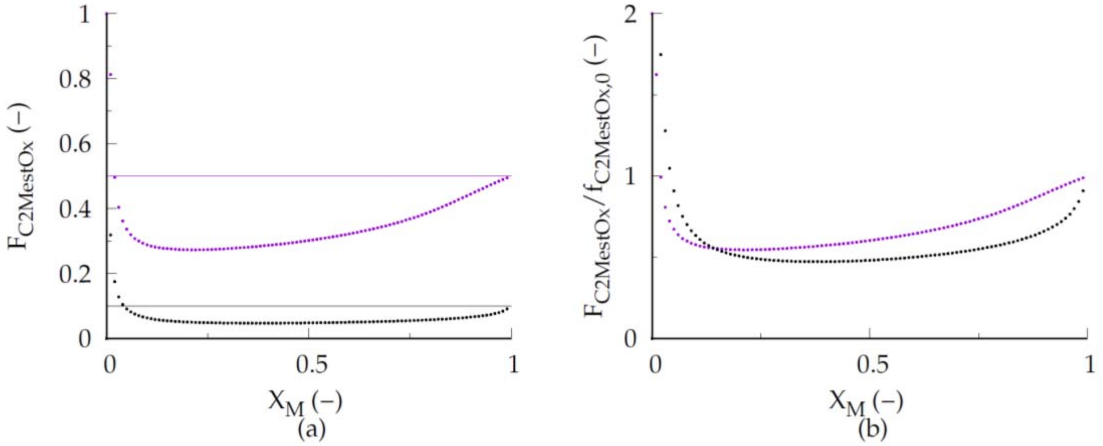

Supplementary Figure 13: Relevance of FUNC-CLDs (part 1). (a) Average cumulative functionalization ($F_{C2MestOx}$) as a function of the overall monomer conversion (X_m); CROP of MeOx and C2MestOX under equimolar conditions (total monomer concentration: 3 mol L^{-1}; solvent acetonitrile; target DP of 100; 140 °C); purple and black dots: initial amount of C2MestOx of 50 and 10 mol %; dashed lines are the desired incorporation according the initial feed; (b) corresponding ratios with target values of 1.

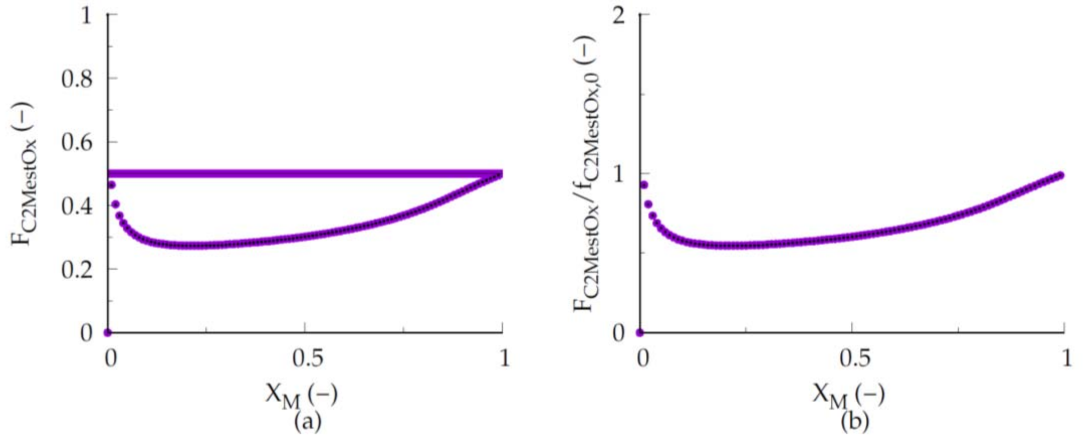

Supplementary Figure 14: Relevance of FUNC-CLDs (part 2); analogous figure as Supplementary Figure 13 (so (a) average cumulative functionalization and (b) the relate normalization) for 50 mol% case displaying the results with and without chain transfer. Identical results indicate the need of FUNC-CLDs as covered in the main text.

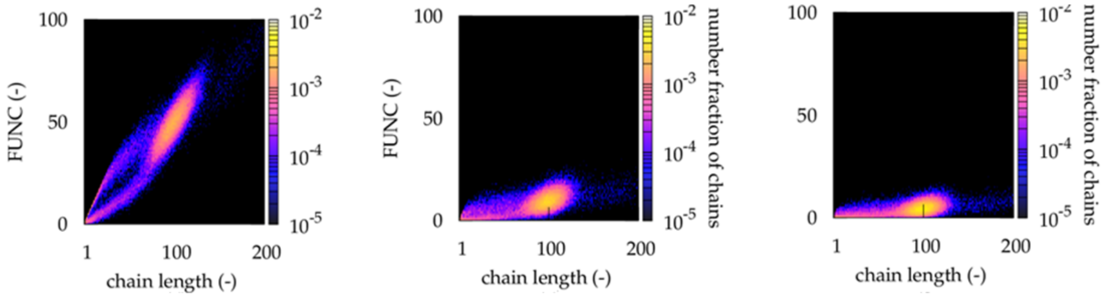

(a) (b) (c)

Supplementary Figure 15: Effect of initial C2MestOx amount ((a) 50, (b) 10, and (c) 5 mol %) on functionalization - chain length distribution (FUNC-CLD). At overall monomer conversion (X_m) of 100% (total monomer concentration: 3 mol L^{-1}; solvent acetonitrile; target DP of 100; 140 °C).

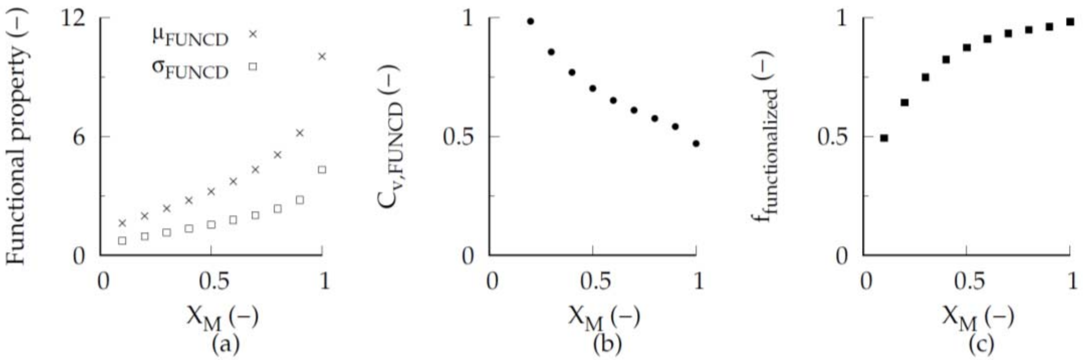

Supplementary Figure 16: Relevance of more than one derived parameter of FUNC-CLD. (a) Mean value and variation for the variance (σ_{FUNCD}) (μ); (b) coefficient of variation (C_v); and (c) fraction of functionalized chains ($f_{functionalized}$) as a function of the overall monomer conversion (X_m); conditions for Figure 8b.

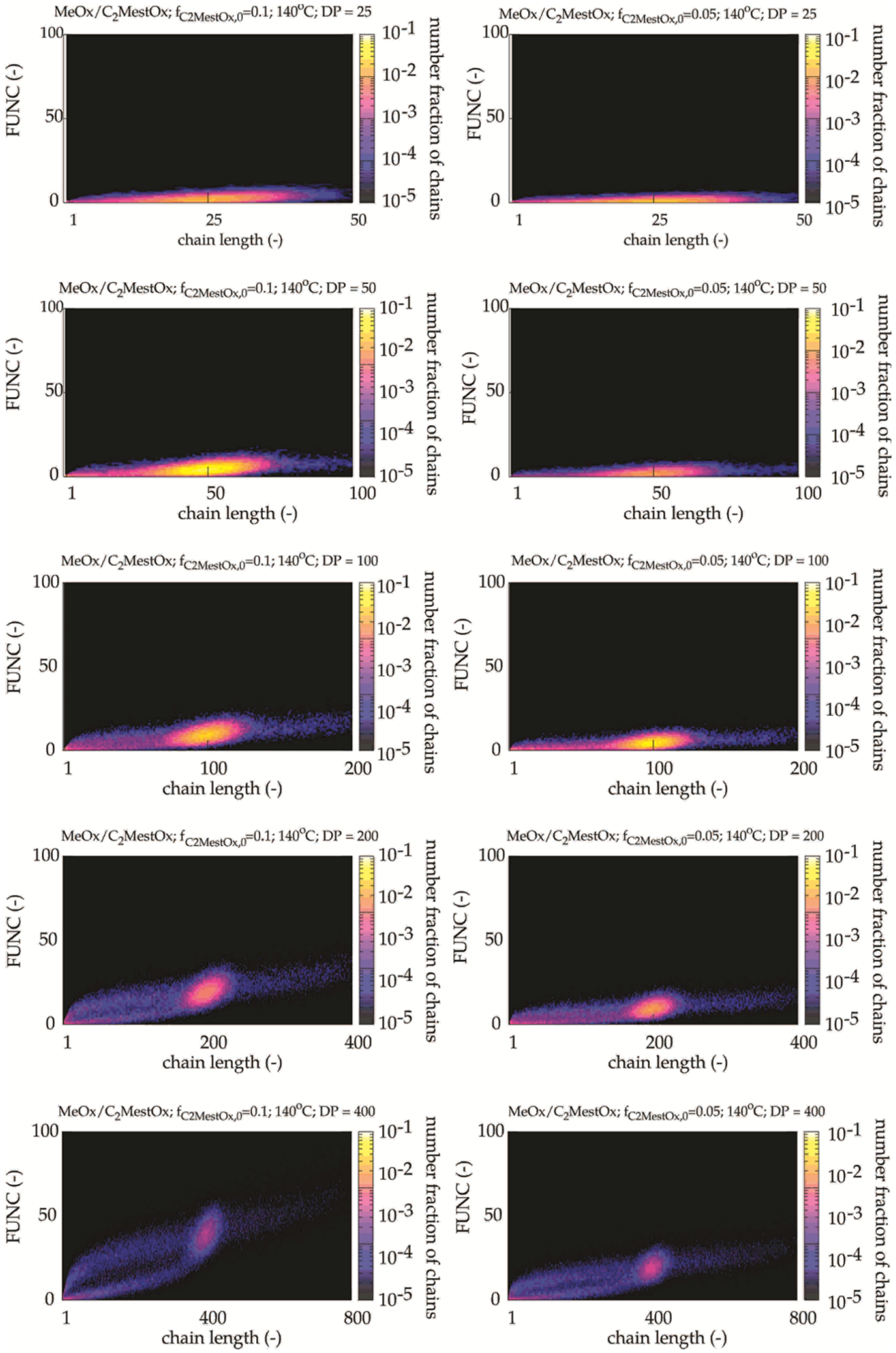

Supplementary Figure 17. Effect of initial functional comonomer amount; columns; 10 and 5 % and target DP (rows; 25, 50, 100, 200, 400) on FUNC-CLD for CROP of MeOx and C2MestOx (total monomer concentration: 3 mol L^{-1}; solvent acetonitrile; Temperature = 140°C; overall monomer conversion (X_m): 100%)

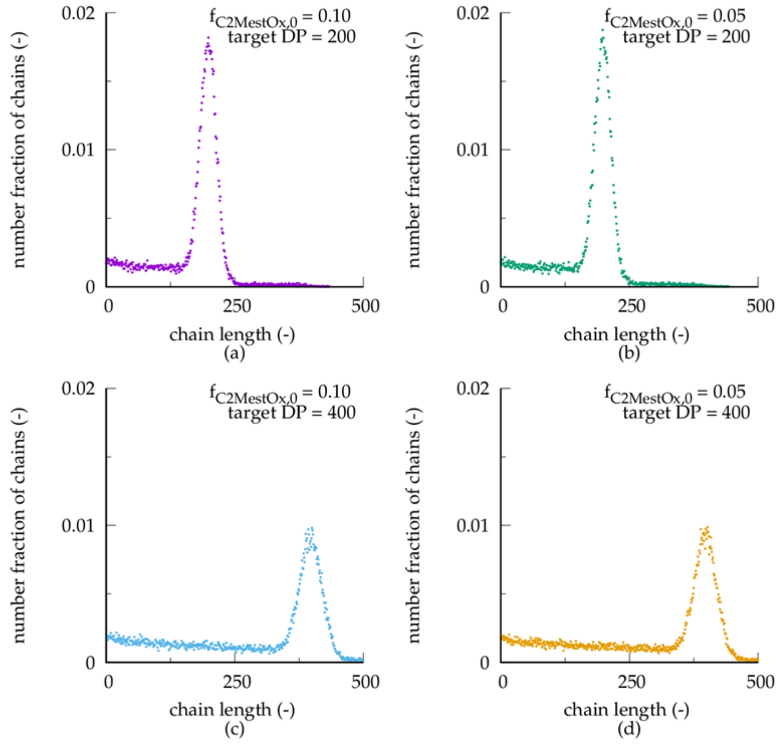

Supplementary Figure 18: Chain length distributions (CLDs) for higher target DPs; T=140°C; comonomer pair MeOx-C2MestOx.

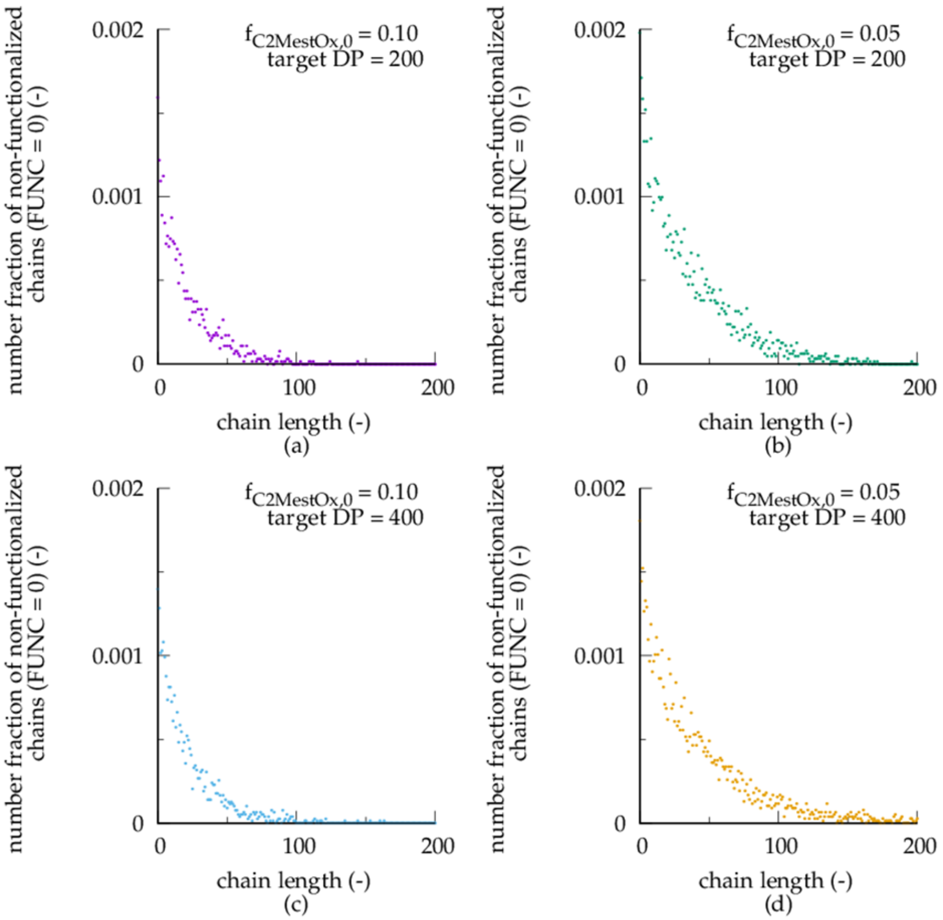

Supplementary Figure 19: The conditional distributions (FUNC=0%; non-functionalized chains) for Supplementary Figure 18; conditional implies that the fractions are directly taken from the latter figure.

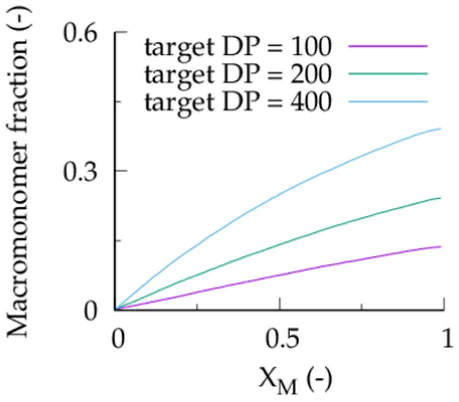

Supplementary Figure 20: Extra information for Figure 10 in the main text related to relevance of chain transfer; first column; 10 mol %; target DPs of 100, 200, 400).

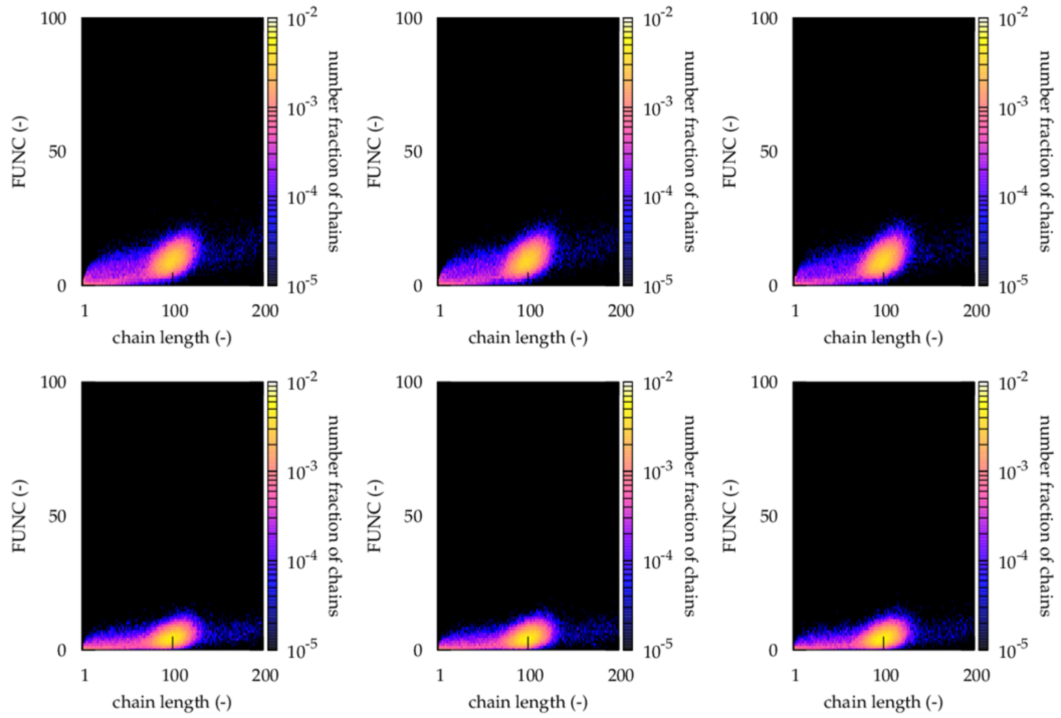

Supplementary Figure 21: Effect of temperature and initial functional monomer amount on the fingerprints for CROP of MeOx and C2MestOx; total monomer concentration: 3 mol L^{-1}; solvent acetonitrile; target DP of 100; overall monomer conversion (X_m)=100%). The subplots are ordered in the same way as Supplementary Table 16 in the main text: $f_{C2MestOx,0}$ = 10 (top), 5 (bottom). Temperature = 140°C (left), 120°C (center), 100°C (right).

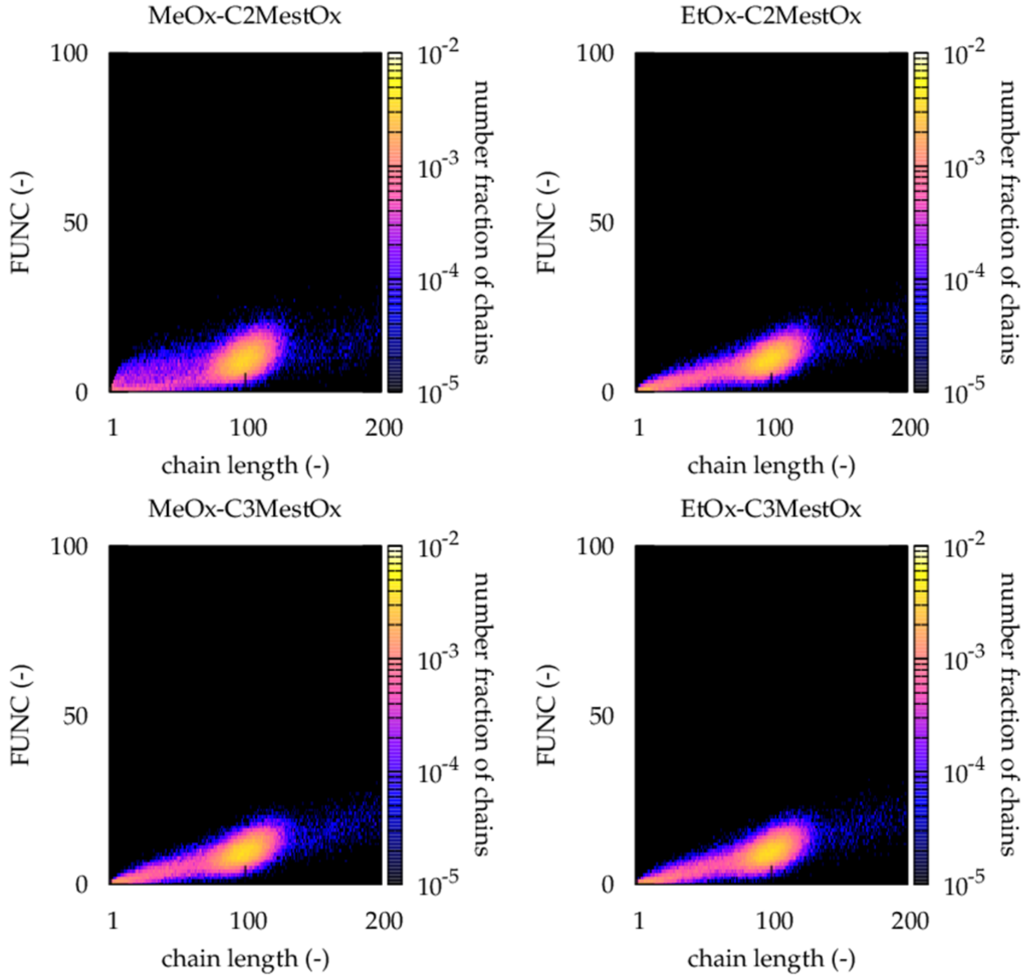

Supplementary Figure 22: FUNC-CLDs for CROP of MeOx-C2MestOx, EtOx-C2MestOx, MeOx-C3MestOx and EtOx-C2MestOx; total monomer concentration: 3 mol L^{-1}; target DP of 100; 10 mol % of methyl ester initially; solvent acetonitrile; overall monomer conversion (X_m)=100%; Temperature = 140°C).

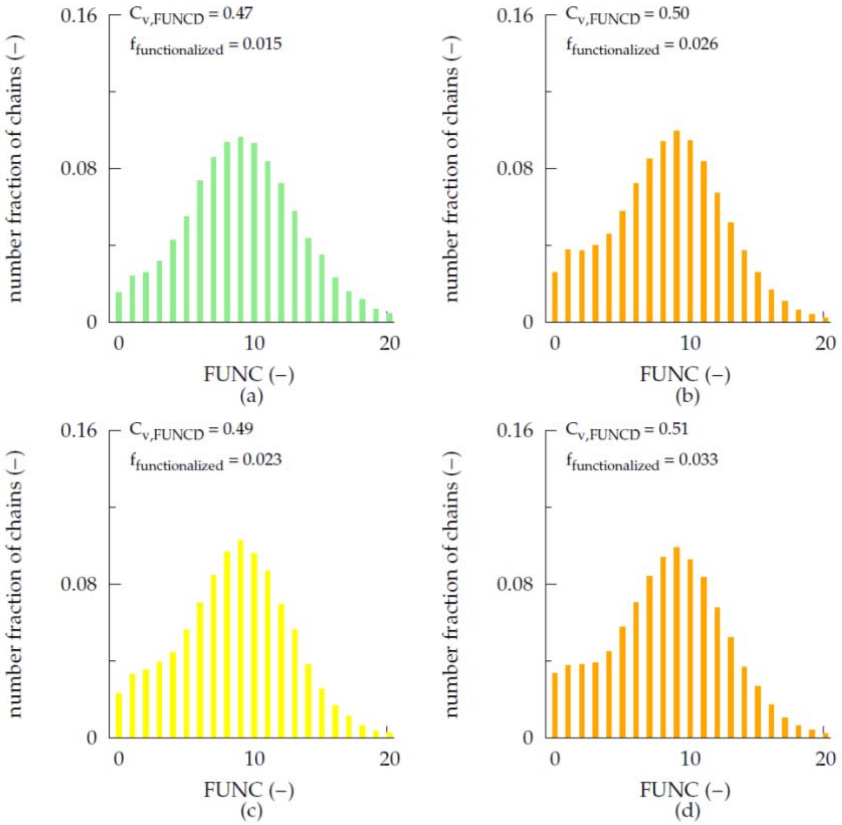

Supplementary Figure 23: Corresponding FUNCDs for Supplementary Figure 22; color coding Figure 7d in the main text; guide of the eye principle.

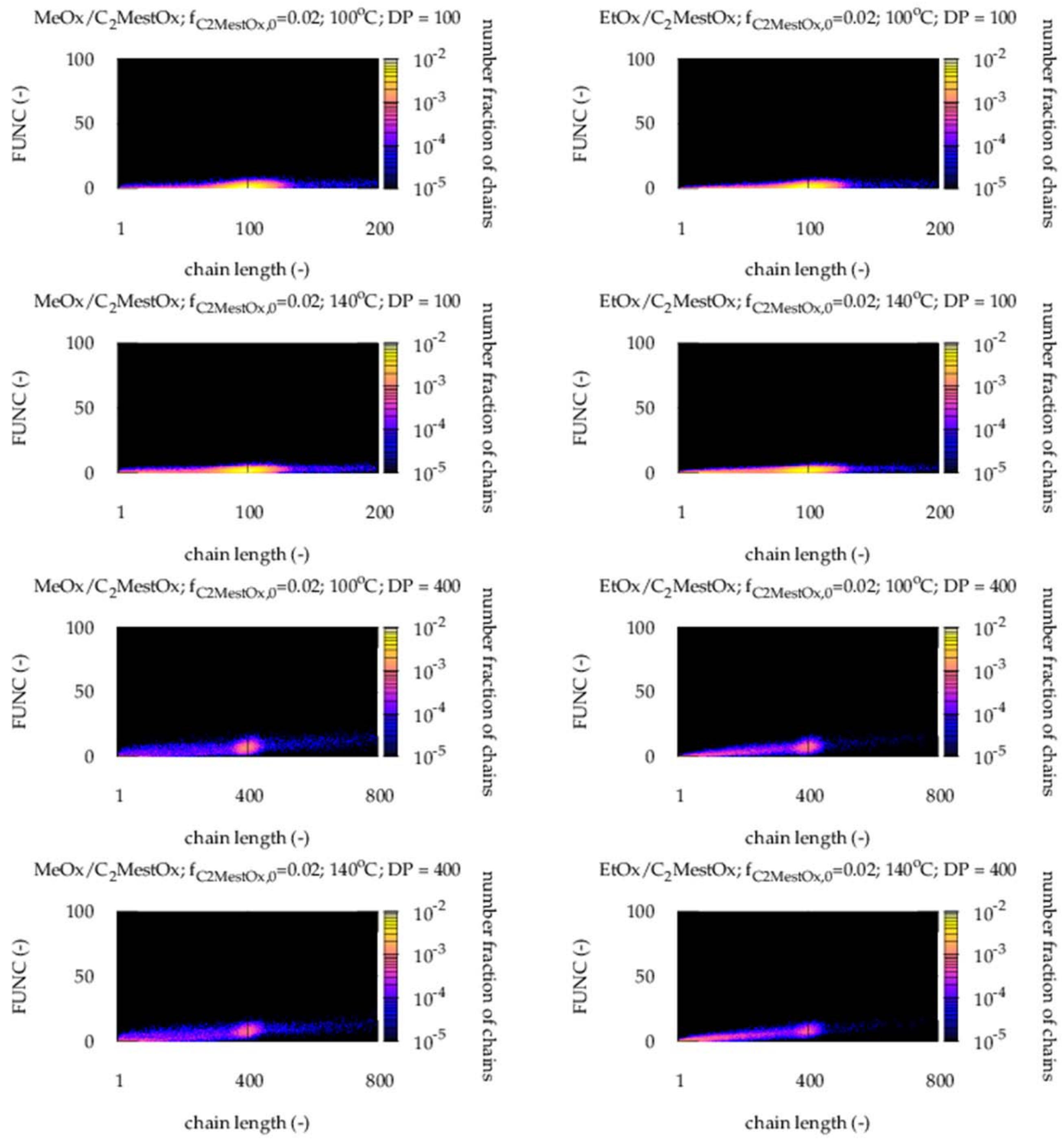

Supplementary Figure 24: FUNC-CLDs for CROP of MeOx-C2MestOx (left column) and EtOx-C2MestOx (right column). Total monomer concentration: 3 mol L^{-1}; 2 mol % of methyl ester initially; solvent acetonitrile; overall monomer conversion (X_m)=100%. Target DP of 100 (first two rows) and 400 (last two rows); Temperature = 100°C (odd rows) and 140°C (even rows).

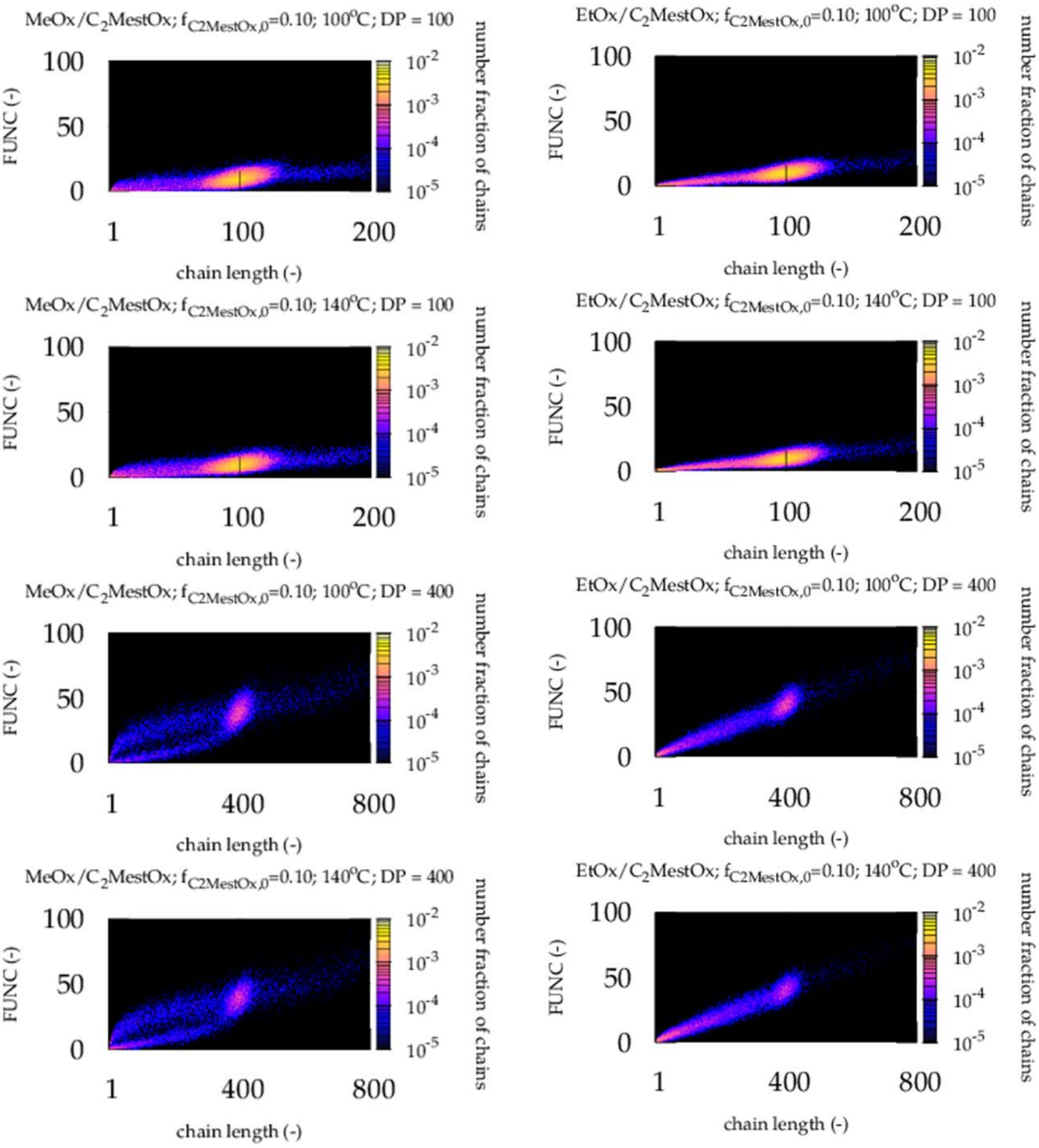

Supplementary Figure 25: FUNC-CLDs for CROP of MeOx-C2MestOx (left column) and EtOx-C2MestOx (right column). Total monomer concentration: 3 mol L^{-1}; 10 mol % of methyl ester initially; solvent acetonitrile; overall monomer conversion (X_m)=100%. Target DP of 100 (first two rows) and 400 (last two rows); Temperature = 100°C (odd rows) and 140°C (even rows).

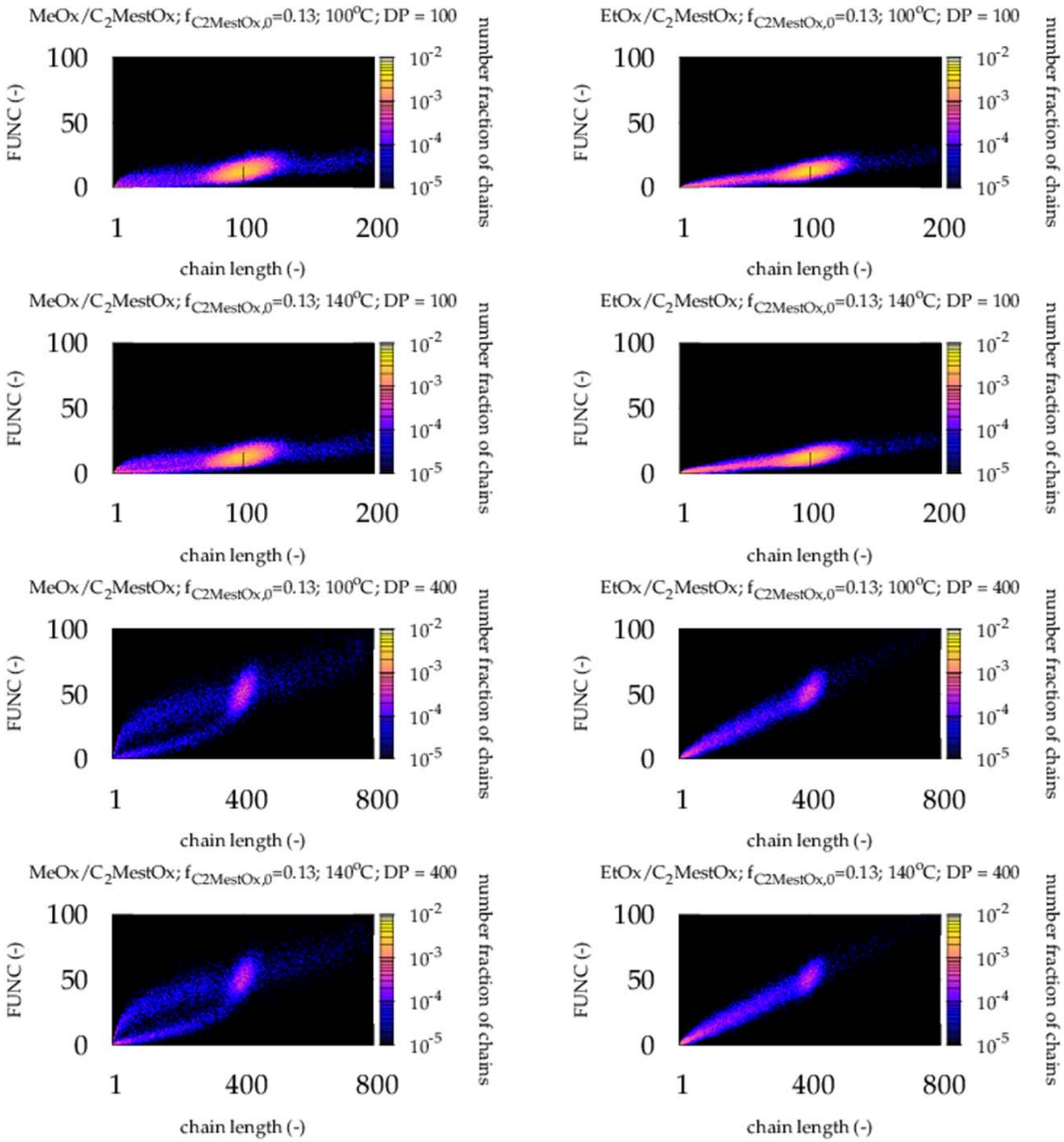

Supplementary Figure 26: FUNC-CLDs for CROP of MeOx-C2MestOx (left column) and EtOx-C2MestOx (right column). Total monomer concentration: 3 mol L^{-1}; 13 mol % of methyl ester initially; solvent acetonitrile; overall monomer conversion (X_m)=100%. Target DP of 100 (first two rows) and 400 (last two rows); Temperature = 100°C (odd rows) and 140°C (even rows).

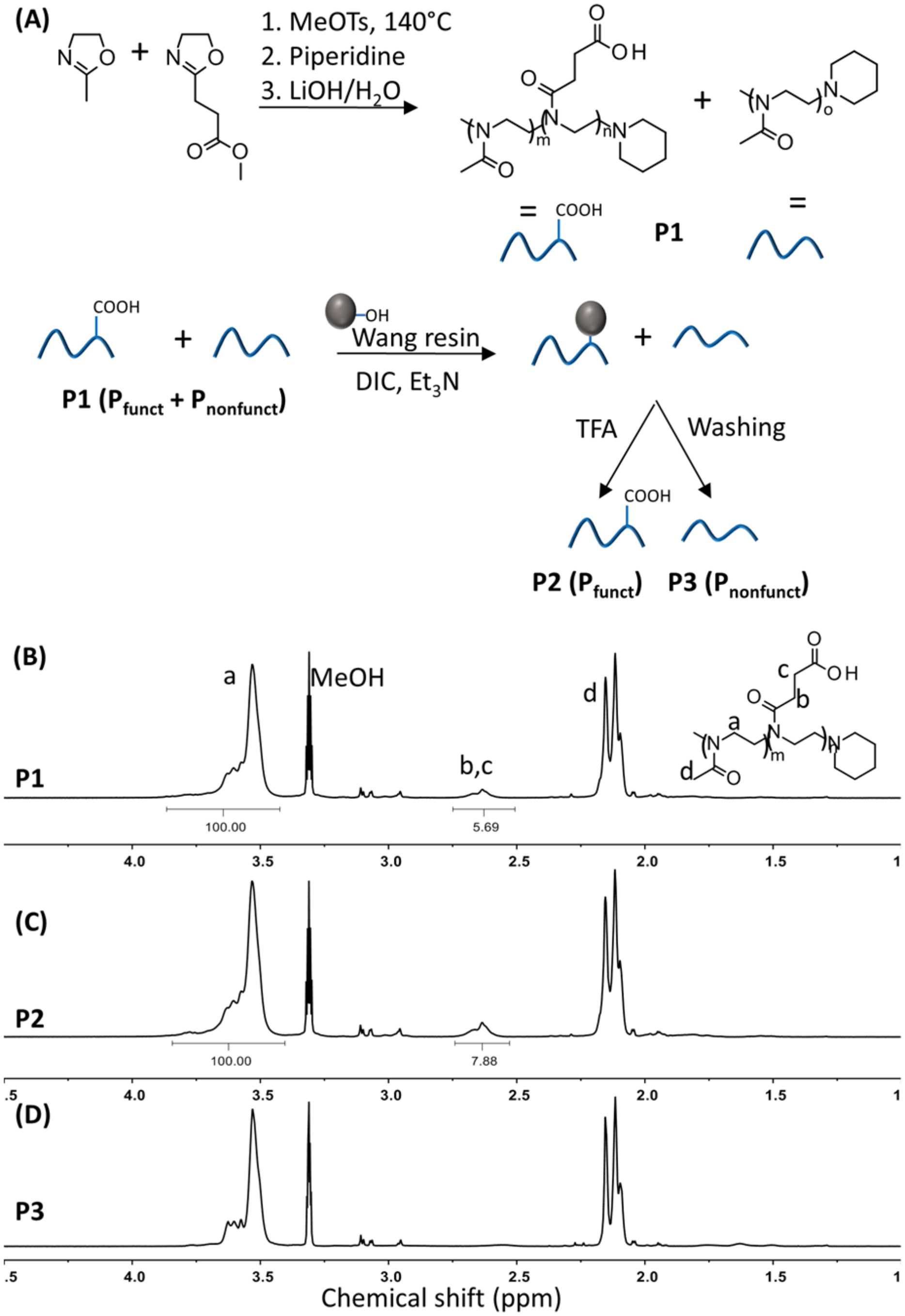

Supplementary Figure 27: Strength of parameter tuning approach; synthetic approach used for the experimental determination of NONFUNC value for PMeOx-PC2MestOx (DP = 25, $f_{C2MestOx,0}$ = 5 mol %) (A). ^{1}H NMR spectra of polymer samples P1 (B), P2 (C) and P3 (D) in CD_3OD. These conditions are selected to maximize the NONFUNC value therefore facilitating the comparison with theory.

Supplementary discussion

X_m is used as short notation in this Supporting Information for the (overall) monomer conversion, hence, in the case of copolymerization of both comonomers together. For the number average chain length the symbol x_n is considered. Target DP stands for target degree of polymerization. Furthermore, the coefficient of variation is denoted as $C_{v,\ FUNCD}$ and the fraction of functionalized chains as $f_{functionalized}$.

Upon the screening of suitable comonomer pairs for the synthesis of well-defined PAOx, attention is focused on the monomer reactivity ratios for propagation, *i.e.* the so-called $r_{(p)}$ values that are defined as the ratio of $k_{p,ii}$ to $k_{p,ij}$ ($i \neq j$). Supplementary Table 7 gives an overview of literature *r* data and additional *r* data determined in the present work by *k*MC (see further in this section), selecting the non-functional monomers PhOx, MeOx, EtOx, 2-*n*-propyl-2-oxazoline (*n*PropOx), *i*PropOx, and 2-*n*-nonyl-2-oxazoline (*n*NonOx), and the functional monomers C2MestOx and C3MestOx, allowing validation of the kinetic parameters used in this work. Most of these data have been obtained at 140°C unless stated otherwise. Since the temperature dependence of the ratios can assumed to be limited,[12] the data in this table can be directly compared, at least to a first approximation.

In Supplementary Table 7, the diagonal elements are by definition unity, as they represent homopropagations. Moving away from the diagonal the reactivity ratios deviate from unity, highlighting the impact of the 2-oxazoline type on the propagation reactivity. It follows that the *r* values are typically higher than unity below the diagonal (favored homo-propagation) and lower than unity above the diagonal (favored cross-propagation). Hence, for a given comonomer pair, it is very likely that one comonomer has a high *r* value and the other one a low *r* value so that a gradient copolymer results in case equimolar conditions are initially selected, which is in agreement with experimental reports.[13, 14, 15] Furthermore, the relevance of the nucleophilicity of the monomer through the presence of an imine group can be

recognized. Upon fixing the macrocation and changing the “cross-monomer” (going from left to right in a row of Supplementary Table 7), the monomer reactivity ratios more or less decrease (more cross-propagation), consistent with the ranking of the these monomers as the nucleophilicity roughly increases from left to right in Supplementary Table 7.[16] The nucleophilicity of EtOx, *n*-PropOx and *n*-NonOx may be considered to be equal based on their very similar reactivity,[17] while C2MestOx and C3MestOx have an apparent lower nucleophilicity based on their slower incorporation in copolymerization with EtOx.[18, 19]

To obtain a complete picture on the suitability of a comonomer pair, the chain transfer reactivities should also be considered. Table 8 lists the monomer reactivity ratios for chain transfer to monomer (r_{ct} values) as introduced and determined in the present work (see Section S3 of the Supporting Information). They are defined as the ratios of the homo- to cross-chain transfer rate coefficients (ratios of $k_{trM,ii}$ to $k_{trM,ij}$). These ratios are important because they allow to assess the potential suppression of chain transfer to one monomer upon addition of a second “cross-monomer”. To the best of our knowledge no such detailed overview is currently available for PAOx synthesis. It follows that the general trends for propagation (*cf.* Table 7; r values) cannot be identified in Table 8, as r_{ct} values both lower and higher than unity appear above and below the diagonal. The effect of increasing nucleophilicity is less straightforward for chain-transfer reactions. Close inspection of Supplementary Table 8 reveals that with the functional comonomer C2MestOx a change of the non-functional monomer from MeOx to EtOx leads to similar r_{ct} values, whereas for C3MestOx a switch from MeOx to EtOx results in an increase of the importance of homo-chain transfer (increasing r_{ct} values).

The macropropagation rate coefficients ($k_{pm,ij}$) have been considered a factor 100 lower than the propagation rate coefficients ($k_{p,ij}$).[1] For simplicity, the propagation reactivity of the mid-chain cationic macrospecies is taken identical as the propagation reactivity of the

conventional end-chain cations. The latter is also acceptable as preliminary simulations showed that the branching amounts are very low, even at very high monomer conversion (< 3 mol%).

The comparison is presented in Supplementary Figure 6-12. An acceptable agreement it obtained in the scope of the present work.

Figure 4c in the main text, which displays the average instantaneous functionalizations ($<F_{\mathrm{C2MestOx,inst}}>$) as a function of the overall monomer conversion (X_m), can be converted in Supplementary Figure13a, which displays the cumulative average functionalization (F_{C2MestOx}), using the initial feed compositions and individual comonomer conversions

$$F_{C2MestOx} = \frac{X_{C2MestOx} f_{C2MestOx,0}}{X_{C2MestOx} f_{C2MestOx,0} + X_{MeOx} f_{MeOx,0}} \tag{1}$$

(X_{MestOx} and X_{C2MestOx}):

Supplementary Figure 13b shows the ratio of $<F_{\mathrm{C2MestOx}}>$ to the targeted value as expressed by the initial feed composition. In agreement with the discussion in the main text, the strongest drifting is obtained for the case with the lower initial functional monomer loading, as larger deviations with respect to reference value of 1 are obtained (black dots vs purple dots).

Supplementary Figure 14 shows that in case chain transfer reactions are formally removed (50 mol% case) the profiles of Supplementary Figure 13 are again obtained. Hence, the use of the comonomer profiles, as typically done, does not allow quantification of the compositional drifting per chain length. Only with the FUNC-CLDs in the main text an interpretation of this drift per chain length becomes possible and copolymer products with and without chain transfer can be properly distinguished.

For the 10 mol % case (140 °C; target DP of 100), the evolution of the mean value (μ_{FUNCD}) and the coefficient of variation ($C_{v,\ FUNCD}$) as a function of the overall monomer conversion (X_m) have been depicted in Supplementary Figure 15 along with the variation for the variance (σ_{FUNCD}) and the fraction

For a DP of 200 and 400, the (number) CLDs are depicted for two initial functional loadings Supplementary Figure 18.

The conditional distribution for the non-functionalized chains (FUNC=0) are given in Supplementary Figure 19. Clearly, the non-functionalized chains are short. By doubling the functional load the maximum chain length also more or less is divided by two.

For the 10 mol % case (140°C, MeOx/C2MestOx), Supplementary Figure 16 shows that a higher target DP the influence of chain transfer is higher, in agreement with the discussion in the main text (Figure 8 in the main text).

The color coding is only included to enable a fast comparison of reaction conditions. Hence, the principle of “guide of the eye” is only followed. However, the key values (transition to green color; 0.5 for $C_{v,FUNCD}$ and 0.05 for $f_{\text{nonfunctionalized}}$) are common values used in the general field of statistics.

A detailed inspection of Figure 7d in the main text allows to deduce the following definitions. An excellent functionalization (dark green color) is defined by a very low $C_{v,FUNCD}$ (≤ 0.4) and NONFUNC (≤ 0.02). A yellow color indicates a good and thus acceptable functionalization quality, with a low $C_{v,FUNCD}$ ($0.4 < C_{v,FUNCD} \leq 0.5$) and NONFUNC ($0.02 <$ NONFUNC ≤ 0.05). In contrast, an orange color corresponds to a FUNCD with only a low $C_{v,FUNCD}$ or only a low NONFUNC, defining a moderate functionalization quality. The transition from dark green to yellow and orange is represented by light green. Finally, a red color is assigned to a FUNCD with a high $C_{v,FUNCD}$ (> 0.50) and a high NONFUNC (> 0.05) or a FUNCD with a

very high $C_{v,FUNCD}$ (> 0.6) or a very high NONFUNC (> 0.08), corresponding to cases of a very bad functionalization quality.

An overview of the reactions for ATRP of styrene (M_1) with small amounts of N-propyl maleimide (M_2) is provided in Supplementary Table 11, with now the terminal unit in superscript to not overload the notation. The ATRP initiator is 1-bromoethyl benzene and the ATRP activator is Cu(I) bromide/4,4'-dinonyl-2,2'-bipyridine.[20] As the polymerization temperature is 110°C thermal self-initiation is accounted for. Since the target degree of polymerization (target DP) is low (100) the impact is however very limited.[21]

The non-ATRP specific parameters, which are the majority of the kinetic parameters in Table Supplementary Table 10, are calculated from Arrhenius parameters reported in literature.[21] The ATRP specific rate coefficients are based on literature data,[21,22] although some tuning of the deactivation rate coefficient for radicals with a styrene terminal unit is performed. Previous work on the related nitroxide mediated polymerization (NMP) technique,[23] in which the focus was on the explicit visualization of monomer sequences and not on the construction of functionalization-chain length distributions (FUNC-CLDs) as covered in the present work, has shown that (de)activation of radicals with a maleimide terminal unit and the related activation step are kinetically insignificant as almost exclusively a styrene monomer addition takes place. Hence, the related rate coefficients can in view of a kinetic description be given a value of 0 L mol^{-1} s^{-1}. The same work has illustrated that a terminal model for the description of the reactivities is sufficient. For termination diffusional limitations are accounted for with the so-called reversible addition fragmentation chain transfer (RAFT)-chain length dependent-termination (RAFT-CLD-T) technique.[24,25]

Model validation is performed based on the experimental data provided in Pfeifer and Lutz,[20] with the summary provided in Supplementary Table 12 (brackets: simulated number). Taking into account experimental error an acceptable agreement between theory and experiment is obtained, highlighting the significance of the FUNC-CLD results in the main text. Overall it can be concluded that for this ATRP case a very limited number of parameters needed to be tuned.

An overview of the reactions for solution ATRP of 2-ethylhexyl acrylate (M_1) and glycidyl methacrylate (M_2) is provided in Supplementary Table 12. The ATRP initiator is methyl 2-bromopropionate and the activator is Cu(I)Cl 2,2-bipyridine. The solvent is toluene and the polymerization temperature is 90°C. A typical reaction ATRP reaction scheme for a copolymerization of an acrylate and a methacrylate is used, neglecting short chain branch formation taking into account its strongly reduced formation under RDRP conditions and the copolymerization nature of the process.[26] An overview of the model parameters in given in Supplementary Table 13, with the propagation kinetic parameters based on pulsed laser polymerization studies and reactivity ratio measurements.[27,28,29] The ATRP activation/deactivation parameters are tuned based on the experimental data in ref. 28, starting from reference values in the systematic study of ref 22 and typical correction factors for secondary and tertiary radicals.[30] Again the RAFT-CLD-T model is used to account for diffusional limitation on termination. Model validation is performed based on literature experimental data.[28] A summary is given in Supplementary Table 14. In the scope of the present work an acceptable agreement between experimental and simulated data is obtained.

Supplementary references

1. Van Steenberge PHM, Verbraeken B, Reyniers M-F, Hoogenboom R, D’hooge DR. Model-Based Visualization and Understanding of Monomer Sequence Formation in Gradient Copoly(2-oxazoline)s On the basis of 2-Methyl-2-oxazoline and 2-Phenyl-2-oxazoline. *Macromolecules* **48**, 7765-7773 (2015).

2. Wiesbrock F*, et al.* Microwave-Assisted Synthesis of a 42-Membered Library of Diblock Copoly(2-oxazoline)s and Chain-Extended Homo Poly(2-oxazoline)s and Their Thermal Characterization. *Macromolecules* **38**, 7957-7966 (2005).

3. Bouten PJM*, et al.* Synthesis of poly(2-oxazoline)s with side chain methyl ester functionalities: Detailed understanding of living copolymerization behavior of methyl ester containing monomers with 2-alkyl-2-oxazolines. *Journal of Polymer Science Part A: Polymer Chemistry* **53**, 2649-2661 (2015).

4. Fijten MWM, Hoogenboom R, Schubert US. Initiator effect on the cationic ring□opening copolymerization of 2□ethyl□2□oxazoline and 2□phenyl□2□oxazoline. *Journal of Polymer Science Part A: Polymer Chemistry* **46**, 4804-4816 (2008).

5. Lambermont□Thijs HML, Jochems MJHC, Hoogenboom R, Schubert US. Synthesis and properties of gradient copolymers based on 2□phenyl□2□oxazoline and 2□nonyl□2□oxazoline. *Journal of Polymer Science Part A: Polymer Chemistry* **47**, 6433-6440 (2009).

6. Park J-S, Kataoka K. Precise Control of Lower Critical Solution Temperature of Thermosensitive Poly(2-isopropyl-2-oxazoline) via Gradient Copolymerization with 2-Ethyl-2-oxazoline as a Hydrophilic Comonomer. *Macromolecules* **39**, 6622-6630 (2006).

7. Park J-S, Kataoka K. Comprehensive and Accurate Control of Thermosensitivity of Poly(2-alkyl-2-oxazoline)s via Well-Defined Gradient or Random Copolymerization. *Macromolecules* **40**, 3599-3609 (2007).

8. Hoogenboom R, Thijs HML, Jochems MJHC, van Lankvelt BM, Fijten MWM, Schubert US. Tuning the LCST of poly(2-oxazoline)s by varying composition and molecular weight: alternatives to poly(N-isopropylacrylamide)? *Chemical Communications*, 5758-5760 (2008).

9. Fijten MWM*, et al.* Synthesis and Structure−Property Relationships of Random and Block Copolymers: A Direct Comparison for Copoly(2-oxazoline)s. *Macromolecules* **40**, 5879-5886 (2007).

10. Richard H, L. THM, M. FMW, M. vLB, S. SU. One□pot synthesis of 2□phenyl□2□oxazoline□containing quasi□diblock copoly(2□oxazoline)s under microwave irradiation. *Journal of Polymer Science Part A: Polymer Chemistry* **45**, 416-422 (2007).

11. Wiesbrock F, Hoogenboom R, Abeln CH, Schubert US. Single-Mode Microwave Ovens as New Reaction Devices: Accelerating the Living Polymerization of 2-Ethyl-2-Oxazoline. *Macromolecular Rapid Communications* **25**, 1895-1899 (2004).

12. Bouten PJM*, et al.* Accelerated living cationic ring-opening polymerization of a methyl ester functionalized 2-oxazoline monomer. *Polymer Chemistry* **6**, 514-518 (2015).

13. Hoogenboom R. Poly(2□oxazoline)s: A Polymer Class with Numerous Potential Applications. *Angewandte Chemie International Edition* **48**, 7978-7994 (2009).

14. Verbraeken B, Monnery BD, Lava K, Hoogenboom R. The chemistry of poly(2-oxazoline)s. *European Polymer Journal* **88**, 451-469 (2017).

15. Verbraeken B, Lava K, Hoogenboom R. Poly(2□oxazoline)s. In: *Encyclopedia of Polymer Science and Technology* (ed^(eds) (2014).

16. Aoi K, Okada M. Polymerization of oxazolines. *Progress in Polymer Science* **21**, 151-208 (1996).

17. Hoogenboom R, Fijten MWM, Thijs HML, van Lankvelt BM, Schubert US. Microwave-assisted synthesis and properties of a series of poly(2-alkyl-2-oxazoline)s. *Designed Monomers and Polymers* **8**, 659-671 (2005).

18. Kobayashi S, Uyama H. Polymerization of cyclic imino ethers: From its discovery to the present state of the art. *Journal of Polymer Science Part A: Polymer Chemistry* **40**, 192-209 (2002).

19. Beck M*, et al.* Polyoxazoline auf fettchemischer Basis. *Die Angewandte Makromolekulare Chemie* **223**, 217-233 (1994).

20. Pfeifer S, Lutz J. Development of a library of N-substituted maleimides for the local functionalization of linear polymer chains. *Chemistry – A European Journal* **14**, 10949-10957 (2008).

21. Fierens SK, D'hooge DR, Van Steenberge PHM, Reyniers MF, Marin GB. MAMA-SG1 initiated nitroxide mediated polymerization of styrene: from Arrhenius parameters to model-based design. *Chemical Engineering Journal* **278**, 407 (2015).

22. Tang W, Kwak Y, Braunecker W, Tsarevsky NV, Coote, ML, Matyjaszeski, K. Understanding atom transfer radical polymerization: effect of ligand and initiator structures on the equilibrium constants. *Journal of the American Chemical Society* **130**, 10702-10713 (2008).

23. Fierens SK, Telitel, S, Van Steenberge PHM., Reyniers MF, Marin GB, Lutz JF, D'hooge DR. Model-based design to push the boundaries of sequence control. *Macromolecules* **49**, 9336-9344 (2016).

24. Russell GT, Barner-Kowollik C Chain-length-dependent termination in radical polymerization: Subtle revolution in tackling a long-standing challenge. *Macromolecules* **34**, 1211-1259 (2009).

25. Derboven P, D'hooge DR, Reyniers MF, Marin GB, Barner-Kowollik C. Model-based design to push the boundaries of sequence control. *Macromolecules* **43**, 492 (2015).

26. Konkolewicz D, Sosnowski S, D'hooge DR, Szymanski R, Reyniers MF, Marin GB, Matyjaszewski, K. Origin of the difference in branching in acrylate polymerization under controlled and free radical conditions: a computational study. *Macromolecules* **44**, 8371 (2011).

27. Junkers T, Schneider-Baumann M, Koo SSP., Castignolles P, Barner-Kowollik C. Determination of propagation rate coefficients for methyl and 2-ethylhexyl acrylate via high frequency PLP-SEC under consideration of the impact of chain branching. *Macromolecules* **43**, 10427-10434 (2010).

28. Dhruba JH, Roy S, Singha, NK. Copper catalyzed atom transfer radical copolymerization of glycidyl methacrylate and 2-ethylhexyl acrylate *Journal of Polymer Science: Part 1: Polymer Chemistry* **47**, 6526-6533 (2009).

29. Jiang JY, Smith LM, Tyrell JH, Coote ML. Pulsed laser polymerisation studies of methyl methacrylate in the presence of AlCl3 and ZnCl2 – evidence of propagation catalysis *Polymer Chemistry* **8**, 5948-5953 (2017).

30. Van Steenberge PMH, Vandenbergh J, Reyniers MF, Junkers T, D'hooge DR, Marin GB. Kinetic Monte Carlo Generation of Complete Electron Spray Ionization Mass Spectra for Acrylate Macromonomer Synthesis *Macromolecules* **50,** 2625-2636 (2017).
